# Distinct transport cycle and lipid regulation of a Mg^2+^-transporting P-type ATPase, MgtA

**DOI:** 10.21203/rs.3.rs-9173029/v1

**Published:** 2026-05-04

**Authors:** Muhammad Bashir Khan, Joseph O. Primeau, Paramita Chaudhuri-Basu, Lucie Bergdoll, Ludovic Renault, Jens Preben Morth, M. Joanne Lemieux, Howard S. Young

**Affiliations:** 1 Department of Biochemistry, University of Alberta, Edmonton, Alberta, Canada; 2 Department of Biotechnology and Biomedicine, Technical University of Denmark, Denmark

## Abstract

P-type ATPases represent an evolutionarily conserved superfamily of ion, lipid, and peptide pumps found across all domains of life. Among the substrates transported by P-type ATPases, Mg^2+^ is of critical importance in bacterial, fungal, and plant cellular homeostasis. A bacterial P-type ATPase found in Gram-negative bacteria, Mg^2+^ transporter A (MgtA), facilitates the transport of Mg^2+^ from the periplasm to the cytoplasm under conditions of Mg^2+^ starvation. MgtA is a cardiolipin-sensitive integral membrane ion-transporter that scavenges Mg^2+^ during bacterial infection and pathogenesis. Here, we determined cryo-EM structures of MgtA capturing three distinct states along the Mg^2+^ transport cycle, including a phosphorylated E2-P intermediate (2.6 Å resolution), an E1-like conformation stabilized by the peptide regulator MgtR (2.7 Å resolution), and an E1-like ATP-bound state (2.8 Å resolution). These three conformations reveal the binding of Mg^2+^ in the transmembrane domain coordinated in a novel site involving Ser^702^ and Asn^706^ on M5, Ser^773^ and Asp^777^ on M7, and Ser^821^ and Thr^824^ on M8. In the E2-P conformation, the phosphate analog BeF_3_ is bound in close proximity to the catalytic aspartate, Asp^361^, suggesting that it represents a covalent aspartylphosphate intermediate. In the presence of AMPPCP, Mg^2+^ remains bound in the transmembrane domain and the ATP analog is bound in a catalytically competent conformation. Overall, the structures reveal distinct steps in the transport cycle of MgtA compared to other P-type ATPases, as well as lipid binding sites that fill gaps in our understanding of transport regulation.

## INTRODUCTION:

Magnesium ions (Mg^2+^) are essential for a wide array of cellular processes, ranging from DNA replication and RNA transcription to energy metabolism and cell growth. Despite its fundamental role in cellular physiology, the diverse mechanisms by which cells maintain Mg^2+^ homeostasis remain poorly understood ^1^. This gap in understanding is due, in part, to the complexity of Mg^2+^ homeostasis and the challenges in studying its transport across cellular membranes. In bacteria, the transport of Mg^2+^ across the plasma membrane is mediated by channels of the CorA and MgtE families and transporters of the CorB/C and MgtA/B families ^2–4^. The MgtA/B family are P-type ATPases mainly found in lower organisms. They belong to the P3B subfamily, which is an orphaned branch of the P-type ATPase superfamily ^5^, though it is nonetheless important as a virulence factor in pathogenic bacteria. MgtA is induced under conditions of limiting Mg^2+^ to maintain intracellular concentrations that allow bacterial survival ^6,7^. Like most P-type ATPases, MgtA has cytoplasmic nucleotide binding (N), phosphorylation (P), and actuator (A) domains, and ten transmembrane helices. The transmembrane helices coordinate the transported ions and counter-ions as MgtA transitions between two main E1 and E2 states. For MgtA, it is proposed that the E1 state has high affinity for cytosolic protons and the E2 state has high affinity for periplasmic Mg^2+^ as the counter-transported ion ^8^.

Despite being a bacterial P3B ATPase, MgtA is similar in topology and sequence to the mammalian pumps including the plasma membrane Na^+^, K^+^-ATPase (NaK) and the sarco-endoplasmic reticulum calcium ATPase (SERCA) (~50% sequence similarity). In the known structures of P-type ATPases, the primary transported ions are better understood than the counter-transported ions, which makes MgtA an interesting target because of the Mg^2+^ counter-transport. SERCA has long served as an archetype for the P-type ATPase superfamily with high-resolution structures of most conformational states in the transport cycle ^9,10^. For SERCA, two Ca^2+^ ions bind to the E1 state and 2–3 H^+^ counter-ions bind to the E2 state. SERCA transports Ca^2+^ ions from the cytoplasm to the lumen of the sarco-endoplasmic reticulum, and counter-transports protons from the lumen to the cytoplasm. By comparison, MgtA counter-transports Mg^2+^ from the periplasmic space to the cytoplasm. Since the main transported ions, Ca^2+^ and Mg^2+^, move in opposite directions, understanding the differences between these bacterial and mammalian pumps will reveal new insights into the transport mechanisms of P-type ATPases. Finally, many P-type ATPases are known to be modulated by factors such as lipids and transmembrane peptides, though the structural underpinnings remain unknown. Recent studies have revealed that MgtA is regulated by cardiolipin ^11^ and small transmembrane peptides ^12–14^.

For these reasons, we determined structures of MgtA by cryogenic electron microscopy (cryo-EM) in the presence of Mg^2+^, phosphate analogue (BeF_3_), and ATP analogue (AMPPCP). Using cryo-EM, we determined the structure of MgtA in E2-P·Mg, E1-like·Mg, and E1·ATP·Mg conformations revealing the novel modes of substrate binding and regulation by cardiolipin. We propose a new model for Mg^2+^ transport by MgtA, which advances our understanding of ion transport mechanisms within the P-type ATPase family and underscores the physiological relevance and therapeutic potential of MgtA in bacterial pathogenesis and anti-microbial resistance.

## RESULTS

**Figure 1:**
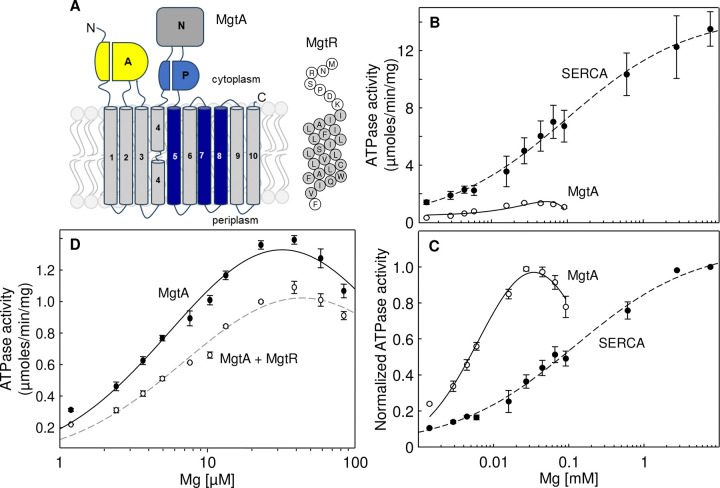
Topology diagram and activity of MgtA. (**A**) Topology diagram of MgtA showing the classical domain architecture of a P-type ATPase with the A (actuator), P (phosphorylation), and N (nucleotide binding) domains, as well as ten transmembrane segments. A topology diagram of MgtR (Salmonella) is also shown. (**B**) Mg^2+^-dependent ATPase activity of MgtA compared to the sarco-endoplasmic reticulum calcium pump SERCA in the detergent-solubilized states. (**C**) Normalized Mg^2+^-dependent ATPase activity of MgtA compared to SERCA. (**D**) Mg^2+^-dependent ATPase activity of MgtA in co-reconstituted membrane vesicles in the absence and presence of MgtR.

MgtA is a Mg^2+^-transporter prevalent in archaea, bacteria, and fungi, with the canonical topology of a P-type ATPase (Figure 1A). MgtA activity peaks at low Mg^2+^ and is reduced by the regulatory peptide MgtR. MgtA from *Lactococcus lactis* was purified in DDM (N-dodecyl-β-D-maltoside) and the Mg^2+^-dependent ATPase activity was measured. This was compared to the Mg^2+^-dependent ATPase activity of SERCA in C_12_E_8_ (octaethylene glycol monododecyl ether). MgtA exhibited peak ATPase activity at ~13 μM Mg^2+^, which was very different from SERCA activity as a function of Mg^2+^ concentration (Figure 1B, C). MgtA activity was also measured in reconstituted proteoliposomes in the absence and presence of the regulatory peptide MgtR. In reconstituted proteoliposomes, the maximal activity of MgtA (V_max_ of 1.3 ± 0.1 μmol/min/mg) was reduced by the regulatory peptide MgtR (V_max_ of 1.0 ± 0.1 μmol/min/mg).

### Structure determination of the MgtA transport cycle

**Extended Data Figure 1:**
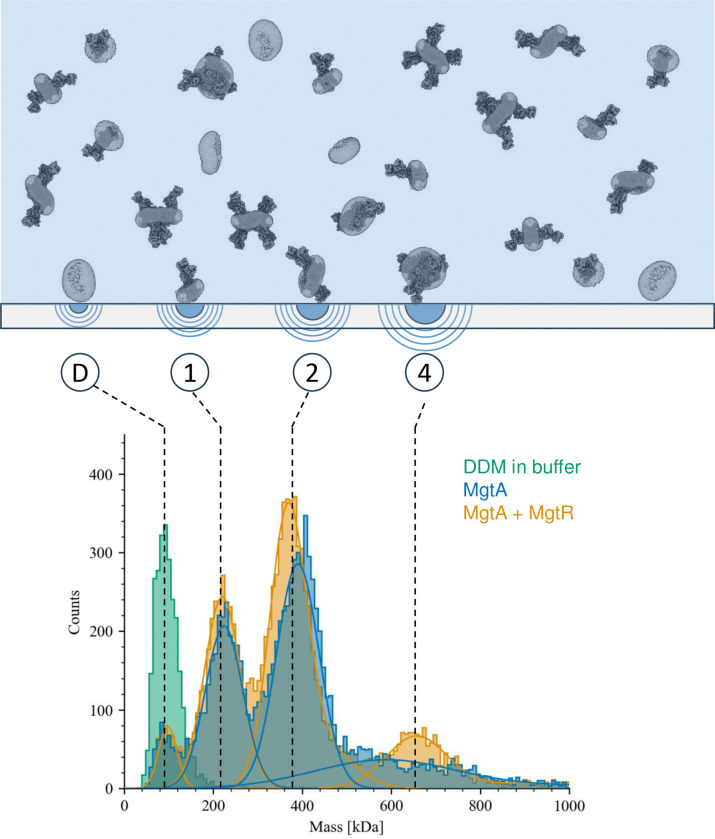
Evaluating sample quality by mass photometry. Histogram of mass photometry counts of MgtA showed clear peaks corresponding to detergent micelle (peak D), along with MgtA in various oligomeric states including monomers (peak 1), dimers (peak 2), and tetramers (peak 4). 20 μL buffer was used to focus the TwoMP optics, 0.2 μL of MgtA was added, and data was recorded for 60 seconds. Bovine serum albumin and apoferritin were used as molecular weight standards in PBS buffer.

Since MgtA transports Mg^2+^ in the same direction that SERCA counter-transports protons, we reasoned that Mg^2+^ must bind to the E2 conformations of MgtA. To stabilize an E2-P state for structural studies, we purified *Lactococcus lactis* MgtA in the presence of Mg^2+^ and the phosphate analogue, beryllium fluoride (BeF_3_^−^). This complex was subjected to structural studies by cryogenic electron microscopy (cryo-EM) and single particle analysis. To evaluate the quality of MgtA samples for vitrification and cryo-EM, we used mass photometry to determine the size and distribution of MgtA in DDM ^15^. Mass photometry of MgtA revealed the presence of four distinct mass profiles indicative of DDM micelles (90 ± 37 kDa), and DDM-solubilized MgtA monomers (225 ± 42 kDa), dimers (390 ± 48 kDa), and tetramers (615 ± 243 kDa) (Extended Data Figure 1). MgtA fractions with well-defined peaks for the oligomeric states and the lowest relative concentration of free detergent micelles were prioritized for vitrification.

**Extended Data Figure 2:**
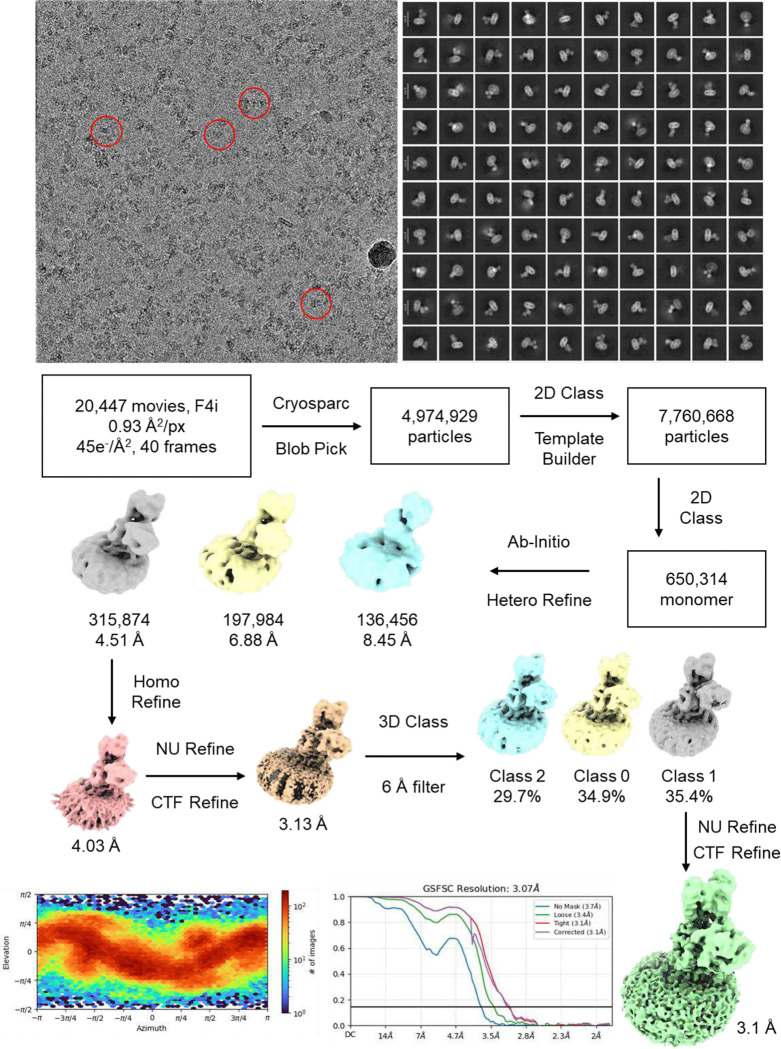

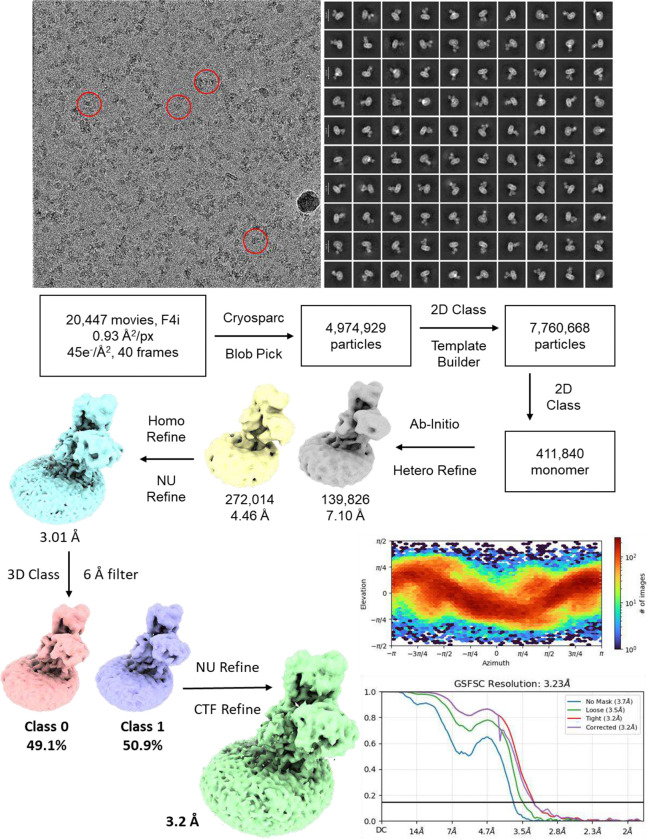

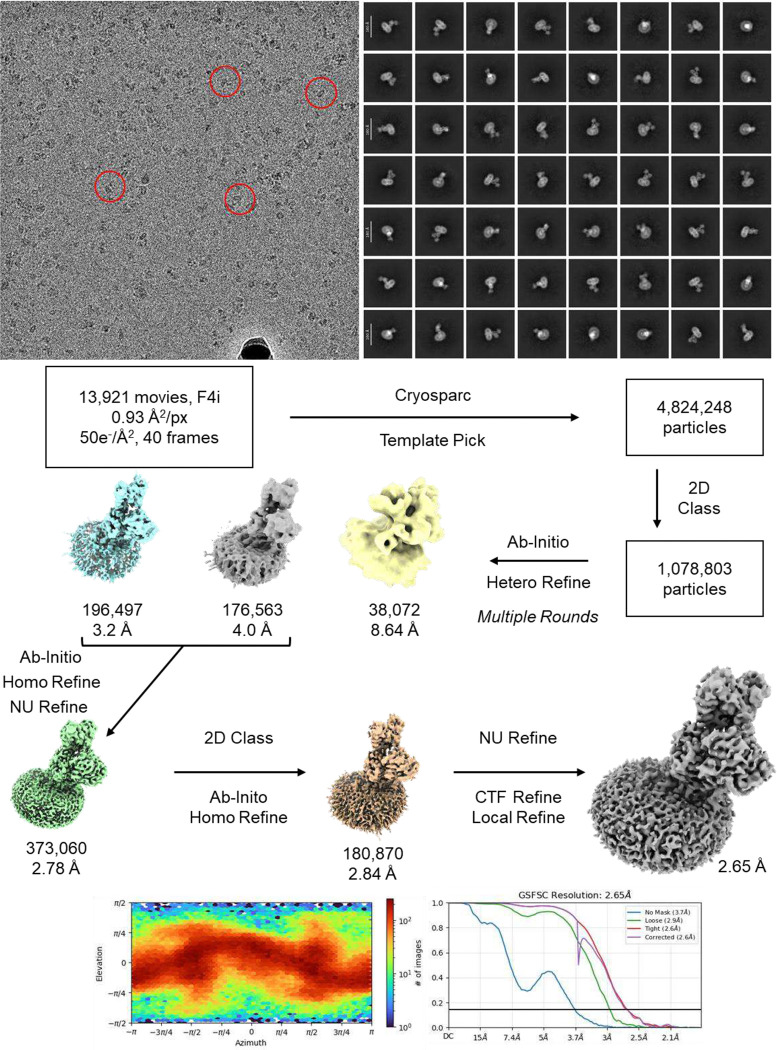

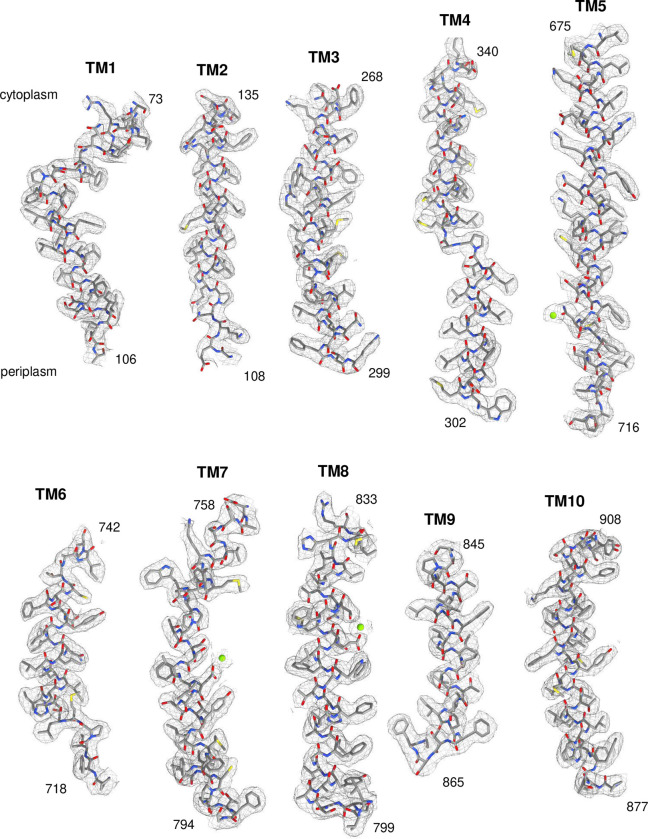

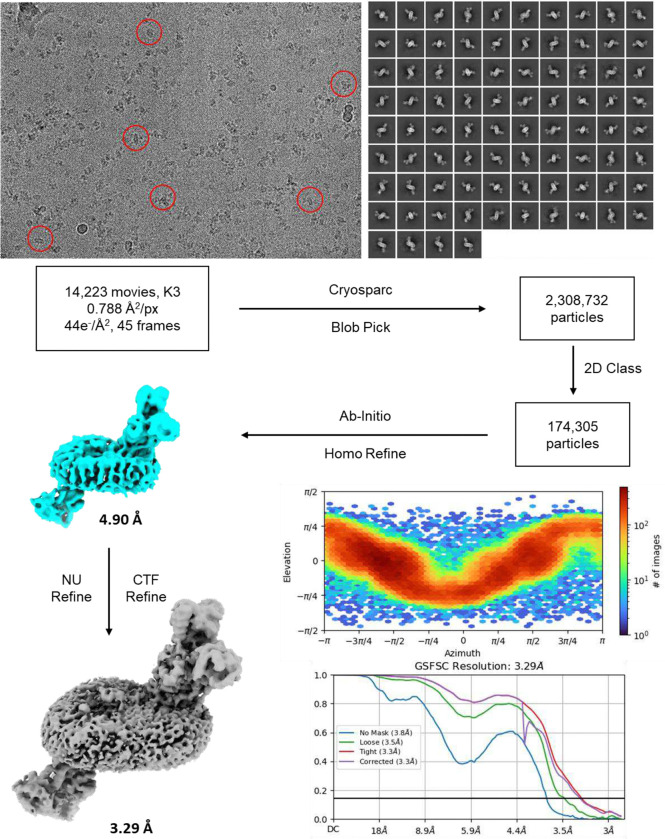

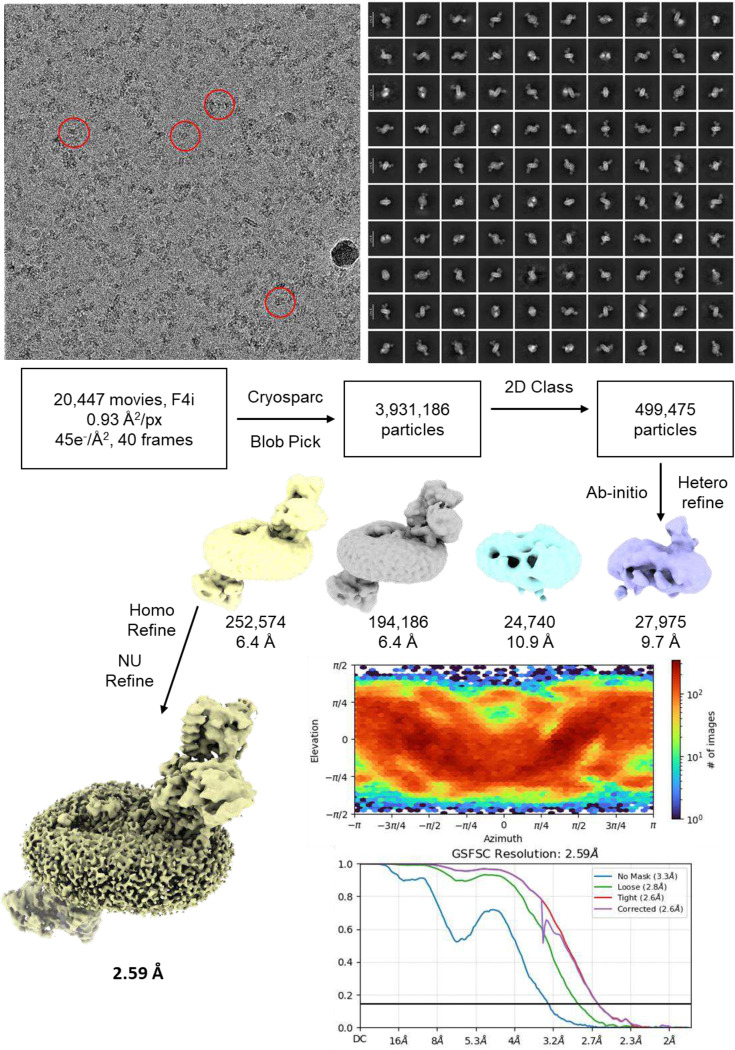

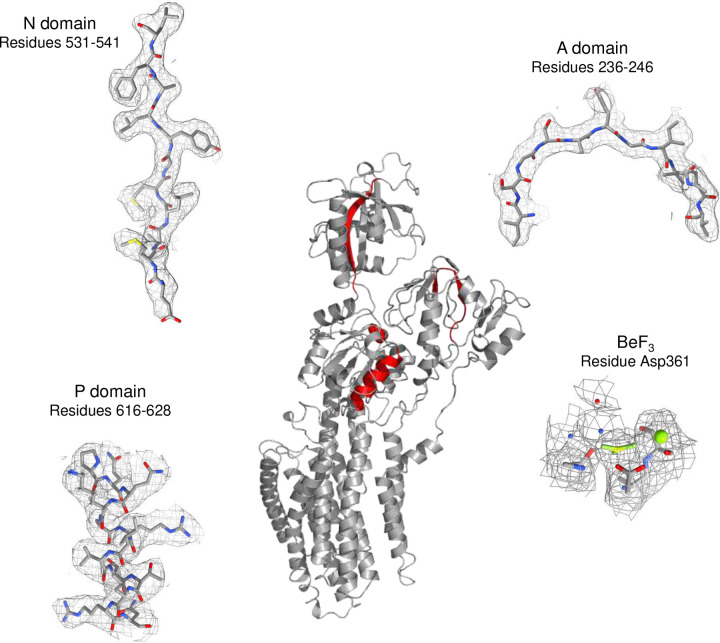

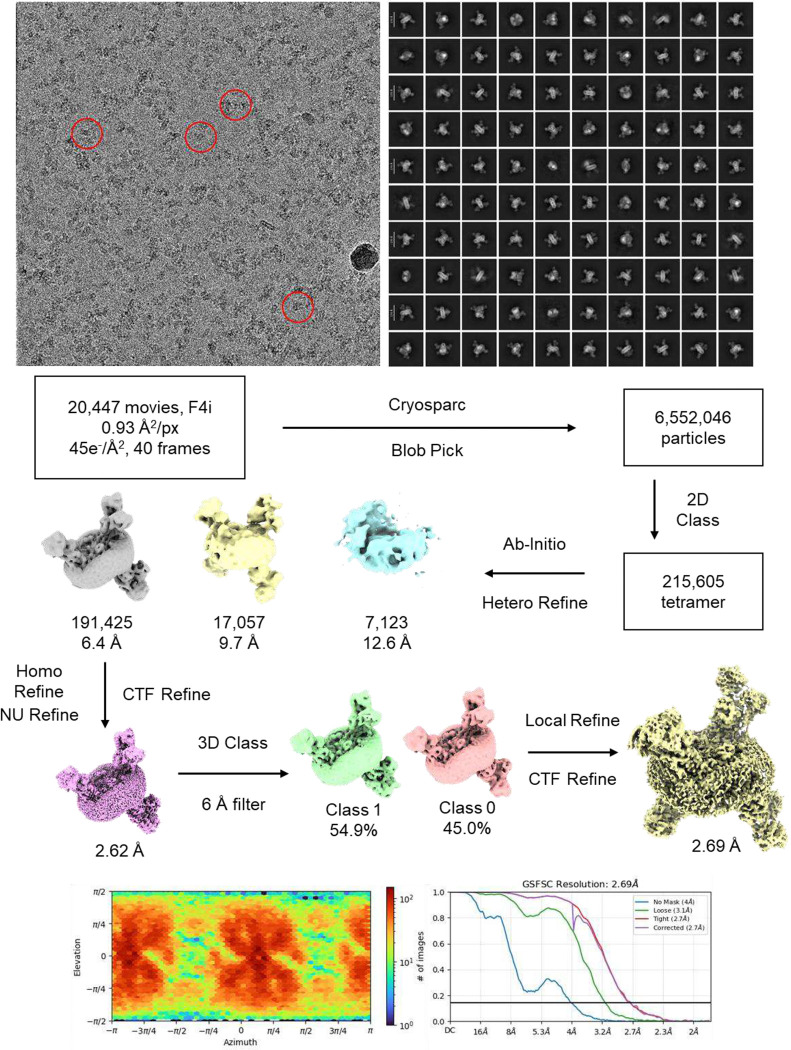

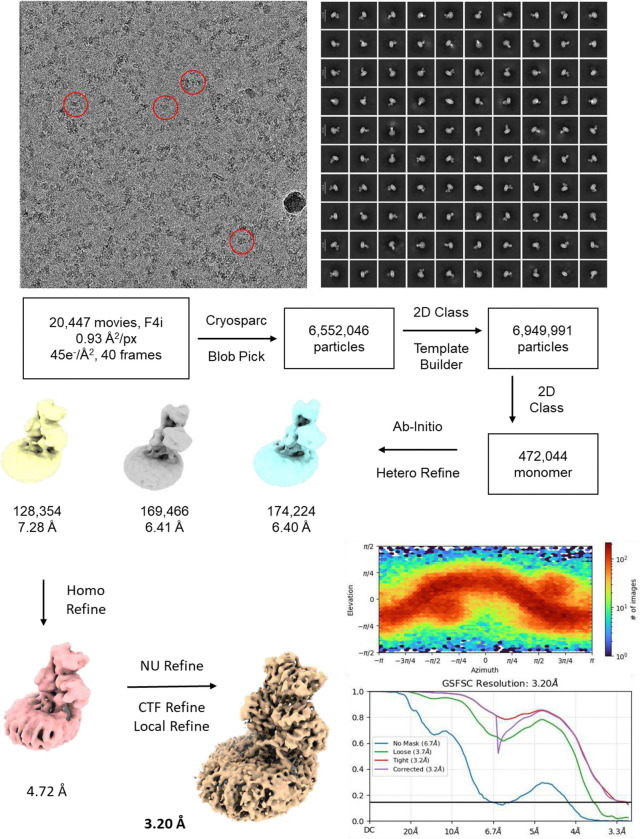

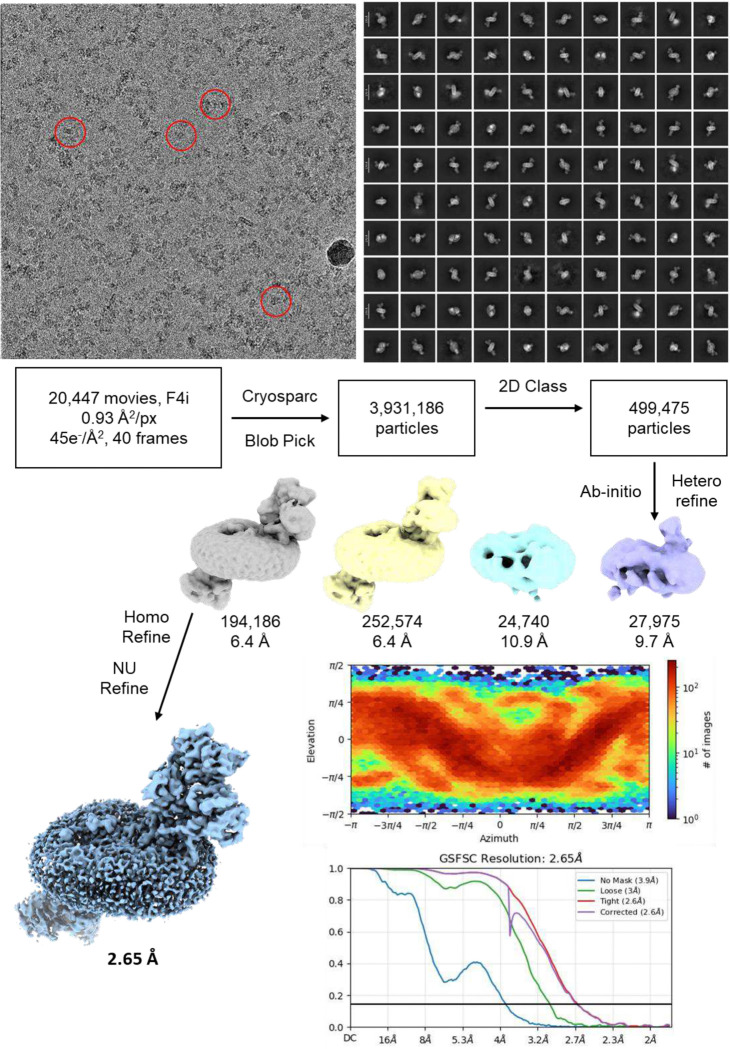

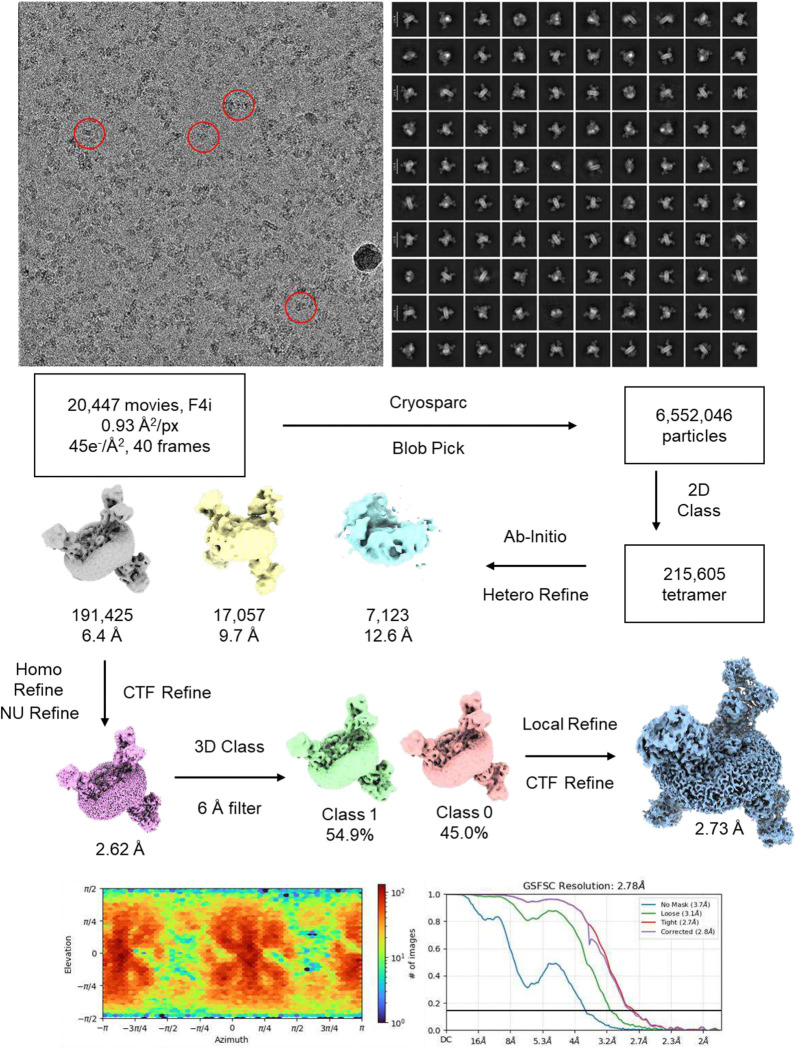

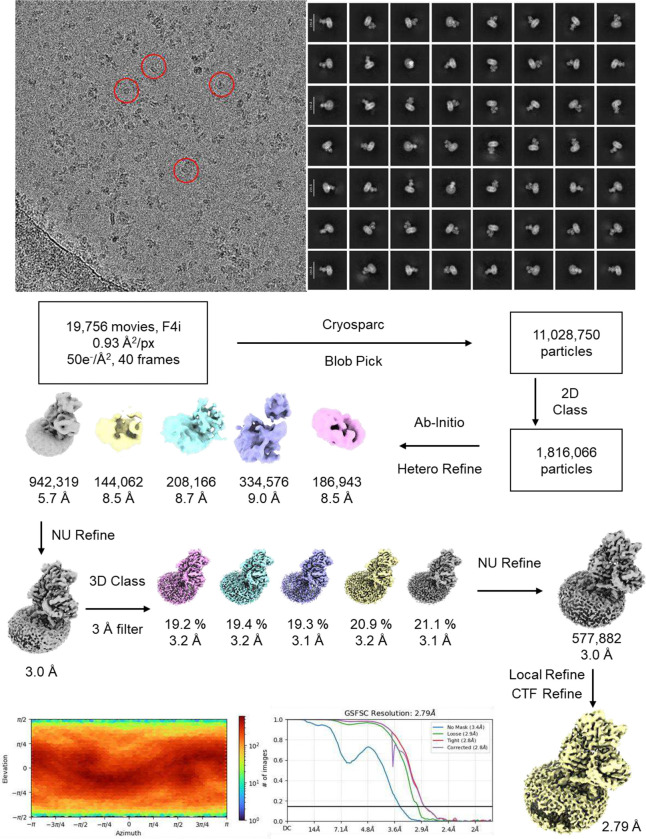
Cryo-EM processing workflow for MgtA in the absence and presence of MgtR. Representative micrograph of the MgtA sample, low-pass filtered to 3 Å. Summary of cryo-EM data processing, showing representative 2D classes, ab initio volumes, and the final reconstruction. Viewing direction distribution plot and Gold Standard Fourier Shell Correlation (GSFSC) as a function of resolution for the map are shown. (**A**) MgtA monomer in the presence of Mg^2+^ and BeF_3_^−^ in E2-P·Mg conformation 1. (**B**) MgtA monomer in the presence of Mg^2+^ and BeF_3_^−^ in E2-P·Mg conformation 2 (change in the position of the A domain). (**C**) MgtA monomer in the presence of Mg^2+^ and BeF_3_^−^ in E2-P·Mg conformation 1. (**D**) Cryo-EM map density associated with the ten transmembrane helices of MgtA (PDB code 9Q6O). The density associated with the bound Mg^2+^ ion is also shown (green sphere). The density map threshold was set to 0.07 in ChimeraX (5σ). The transmembrane segments correspond to MgtA model residues 73–106 (TM1), 108–135 (TM2), 268–299 (TM3), 302–340 (TM4), 675–716 (TM5), 718–742 (TM6), 758–794 (TM7), 799–833 (TM8), 845–865 (TM9), and 877–908 (TM10). (**E**) MgtA dimer in the presence of Mg^2+^ and BeF_3_^−^ in E2-P·Mg conformation. (**F**) MgtA dimer in the presence of Mg^2+^, BeF_3_^−^, and MgtR in E2-P·Mg conformation. (**G**) Cryo-EM map density associated with regions of MgtA cytoplasmic domains (PDB code 9N5J). A ribbon diagram of MgtA is shown with the regions of the map in red. Density associated with the N domain (residues 531–541), the P domain (residues 616–628), the A domain (residues 236–246), and the bound BeF_3_ are shown (Asp361). (**H**) MgtA tetramer in the presence of Mg^2+^ and BeF_3_^−^, and MgtR in E2-P·Mg conformation. (**I**) MgtA monomer in the presence of Mg^2+^, BeF_3_^−^, and MgtR in E1-like·Mg conformation. The density map threshold was set to 0.07 in ChimeraX (5σ). (**J**) MgtA dimer in the presence of Mg^2+^, BeF_3_^−^, and MgtR in mixed E2-P·Mg and E1-like·Mg conformations. (**K**) MgtA tetramer in the presence of Mg^2+^, BeF_3_^−^, and MgtR in mixed E2-P·Mg and E1-like·Mg conformations. (**L**) MgtA monomer in the presence of Mg^2+^ and APMPCP in E1-like·ATP·Mg conformation.

**Figure 2:**
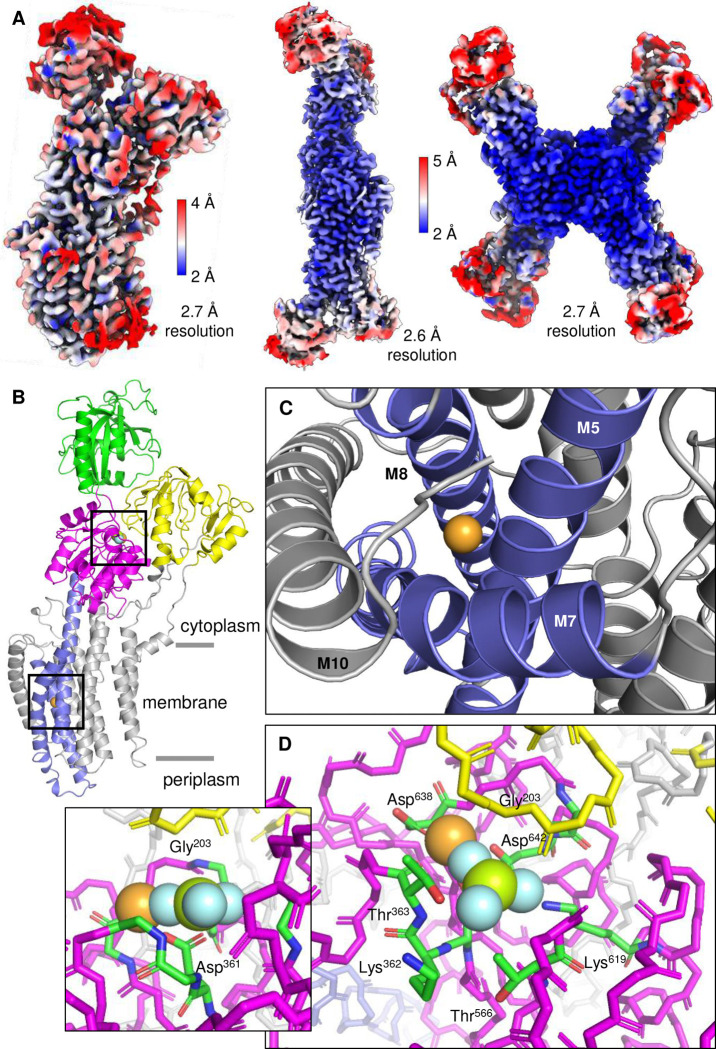
Cryo-EM reconstructions of MgtA in the E2-P·Mg state in the presence of Mg^2+^ and phosphate analogue, BeF_3_^−^. (**A**) Cryo-EM maps colored according to estimated resolution for a monomer, antiparallel dimer, and antiparallel tetramer. (**B**) Cartoon diagram of MgtA in the E2-P·Mg state colored according to the A (yellow), P (magenta), N (green), and transmembrane segments M5, M7, and M8 (blue). Based on PDB code 9Q6O, and consistent with PDB codes 9MQM, 9MT7, 9BYB, 9N5J, and 9Q1E (Table 1). The orientation shown in the ribbon diagram is with the cytoplasm on top and the periplasm on the bottom, which is the opposite orientation normally shown for bacterial cell membrane proteins. However, to facilitate comparison with the extensive literature on other P-type ATPase such as SERCA, this orientation was chosen. (**C**) A Mg^2+^ ion was identified in the transmembrane domain coordinated by transmembrane helices M5, M7, and M8. (**D**) The phosphate analogue BeF_3_^−^ and Mg^2+^ was found coordinated by the P (Asp^361^) and A (Gly^203^) domains. The proximity between the phosphate analogue and the catalytic aspartate, Asp^361^, suggests that the structure reflects an aspartyl-phosphate form of MgtA. Density map threshold was set to 0.1 in ChimeraX (6σ).

**Figure 3:**
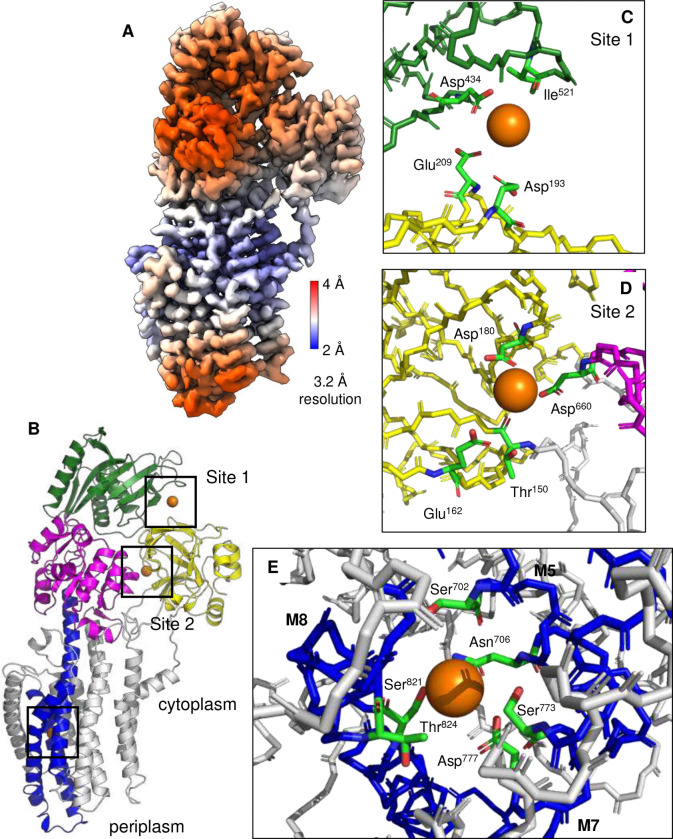
Cryo-EM reconstruction of MgtA in the E1-like·Mg state observed in the presence of MgtR. (**A**) Cryo-EM map colored according to estimated resolution for a monomer. A mixed antiparallel dimer (E2-P·Mg and E1-like·Mg conformations) and mixed anti-parallel tetramer (three E2-P·Mg and one E1-like·Mg conformations) were also reconstructed (not shown). (B) Cartoon diagram of MgtA in the E1-like·Mg state colored according to the A (yellow), P (magenta), N (green), and transmembrane segments M5, M7, and M8 (blue). Based on PDB codes 9ME9, 9N3V, and 9NHZ (Table 1). Three Mg^2+^ ions were identified in the cryo-EM maps. (**C**) Site 1 Mg^2+^ was found coordinated between the N and A domains. (**D**) Site 2 Mg^2+^ was found coordinated between the A and P domains. (**E**) The third Mg^2+^ ion was found coordinated by six residues in the transmembrane domain including two each from M5 (Ser^702^ and Asn^706^), M7 (Ser^773^ and Asp^777^), and M8 (Ser^821^ and Thr^824^). Density map threshold was set to 0.3 in ChimeraX (12σ).

Frozen-hydrated samples were prepared for *L. lactis* MgtA in the presence of Mg^2+^ and BeF_3_^−^, in the absence and presence of the regulatory peptide MgtR. Cryo-EM data collection provided dose-fractionated movies of MgtA embedded in a micelle with canonical features of a P-type ATPase with particles that ranged between 130–220 Å diameter (Extended Data Figure 2). Two-dimensional class averages revealed clear structural architecture of MgtA, including the appearance of transmembrane densities consistent with α-helices and several oligomeric states, including a monomer, anti-parallel dimer, and an anti-parallel tetramer. Three-dimensional (3D) reconstruction of MgtA revealed high-resolution maps of MgtA as a monomer, dimer, and tetramer. We did not observe the MgtA dimer that has been reported previously ^16^. All reconstructed maps had evidence of annular lipids, consistent with cardiolipin molecules lining the transmembrane domain and the dimer and tetramer interfaces. The final maps revealed two main conformations of MgtA, an E2-P conformation with bound Mg^2+^ and BeF_3_^−^ and an E1-like conformation with bound Mg^2+^ that was only found in the presence of MgtR. The E2-P monomer reached an average resolution of 2.7 Å with a local resolution estimate of ~2.2 Å in the transmembrane domain (Figure 2). The E1-like monomer reached an average resolution of 3.2 Å with a local resolution estimate of ~2.2 Å in the transmembrane domain (Figure 3). The lower resolution areas of the maps coincided with the mobile cytoplasmic domains. Nonetheless, the quality of the maps was sufficient to build complete models of MgtA and to identify bound substrates, lipids, and detergents (Table 1).

The dimers and tetramers of MgtA were reconstructed to significantly higher resolution, with local resolution estimates ranging from 1.9 Å in the core of the protein and the transmembrane domain (tetramer) to 3.5 Å in the cytoplasmic domains (dimer). These oligomers consisted of E2-P conformations of MgtA in the absence of MgtR and mixtures of E2-P and E1-like conformations in the presence of MgtR. The E2-P dimer was reconstructed to a resolution of 2.6 Å and the tetramer to a resolution of 2.7 Å (Figure 2 and Table 1). Helix-helix interactions between transmembrane segments M7 and M10 stabilized the antiparallel dimers and tetramers. Both maps were sufficient to generate a complete model of MgtA, identifying a Mg^2+^ ion coordinated in the transmembrane domain, as well as BeF_3_^−^ in the phosphorylation domain and annular cardiolipin lipids. Notably, the tetramers have a lipid core that fills a 16.5 Å gap created by the interaction of dimers in the tetrameric arrangement (discussed below). Mixed E2-P and El-like conformations were also observed in an antiparallel dimer and tetramer (2.7 Å resolution; Table 1). The tetramer contained three E2-P conformations and one E1-like conformation of MgtA.

### E2-P·Mg, a new conformation of MgtA

High-resolution cryo-EM reconstructions facilitated identification of substrates and Mg^2+^-binding sites within the maps. The E2-P conformation of MgtA contained additional densities that were consistent with two bound Mg^2+^ ions, one in the transmembrane domain and a second one coordinated with the phosphate analogue, BeF_3_^−^, in the ATP binding pocket (Figure 2). A Mg^2+^ ion bound in the transmembrane domain was coordinated by residues on transmembrane segments M5, M7, and M8. The involvement of these transmembrane helices appears to be unique among the P-type ATPases, though it is close to the third sodium ion binding site in the Na^+^, K^+^-ATPase ^17^. In both cases, M7 curves away from the ion binding site on the cytoplasmic side of the membrane, which contributes to a more open arrangement of the helices on the cytoplasmic side of the bound Mg^2+^ ion (Figure 2C). The phosphate analogue, beryllium fluoride (BeF_3_^−^), was bound above the catalytic aspartate residue, Asp^361^, in the P domain. The beryllium atom is close to the carbonyl and hydroxyl oxygen atoms of Asp^361^ as expected for a typical member of the P-type ATPase family (Figure 2D; 1.8 Å and 2.8 Å, respectively), suggesting that it represents an aspartyl-phosphate intermediate of MgtA. The residue Gly^203^ of the conserved TGES motif in the A domain is positioned above the BeF_3_^−^, creating space for phosphate binding and coordination by P domain residues Lys^362^, Thr^363^, Thr^566^, and Lys^619^. A Mg^2+^ ion is coordinated with BeF_3_^−^ and the two closest residues Asp^638^ and Thr^363^ of the P domain.

**Extended Data Figure 3:**
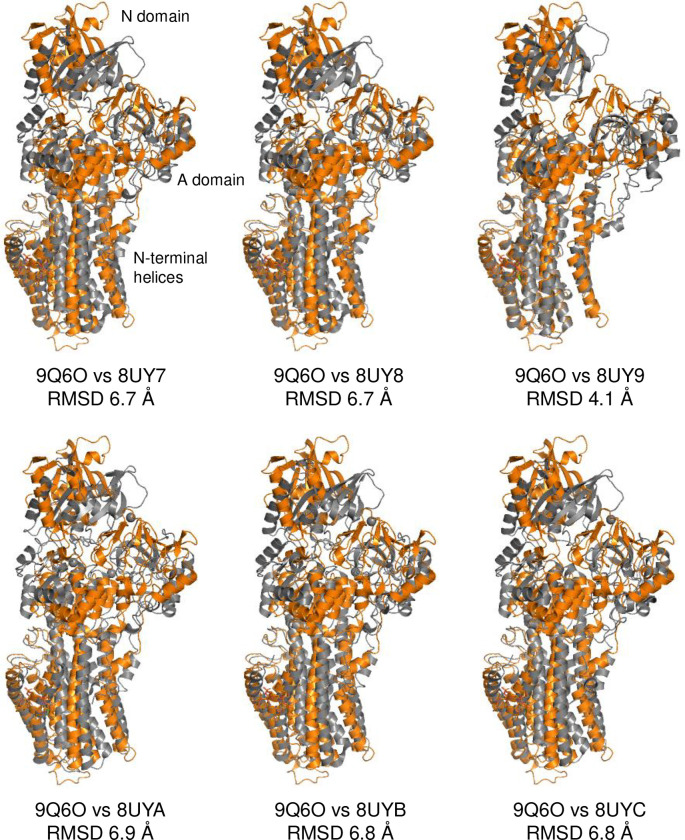
Alignment of the E2-P·Mg conformation of MgtA (orange; PDB code 9Q6O) with previously reported structures of E. coli MgtA (grey (16)). Notice the different positions of the A and N domains, as well as the N-terminal transmembrane helices.

Structural alignment of the E2-P conformation of MgtA with multiple E2-P (BeF_3_^−^) conformations of SERCA (PDB codes 3AR9, 2ZBE, 3B9B, 5A3R, 2ZBF) and Na^+^, K^+^-ATPase (PDB code 7D91) resulted in root-mean-square deviations (RMSD) of ~4 Å across all structures. Selective alignment of the P domains resulted in an RMSD of ~0.8 Å across all structures. Thus, the coordination of BeF_3_^−^ by MgtA is similar to the calcium-free E2-P conformation of SERCA (e.g. PDB code 5A3R; P-domain RMSD 0.73 Å), suggesting that it represents a genuine E2-P conformation of MgtA. The E2-P conformation of MgtA identified here (Figure 2) is distinct from previous structures of MgtA (Extended Data Figure 3 ^16^).

### E1-like·Mg conformation of MgtA

**Extended Data Figure 4:**
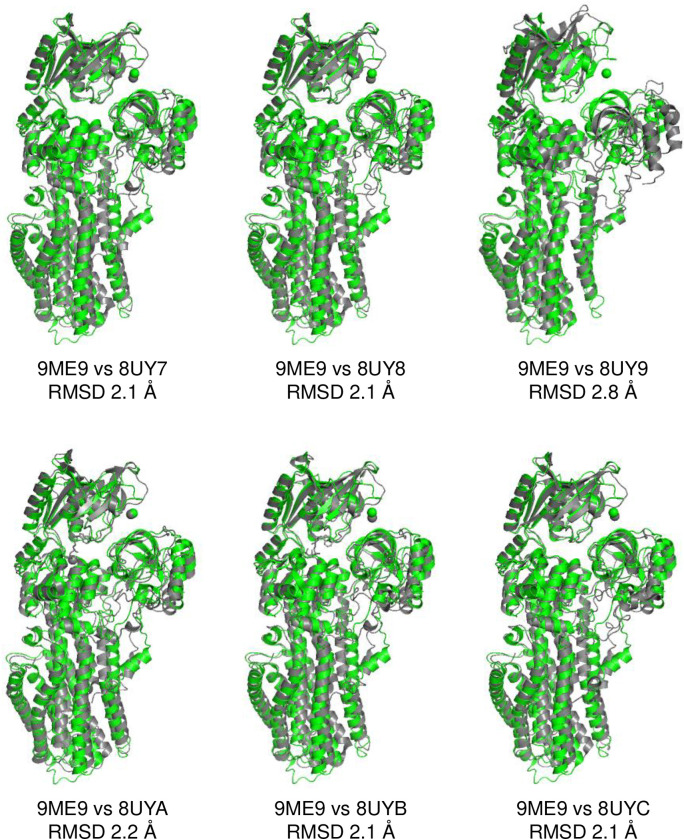
Alignment of the E1-like·Mg conformation of MgtA (orange; PDB code 9ME9) with previously reported structures of E. coli MgtA (grey (16)). The structures are much more similar, though there are differences in the positions of transmembrane segments M1-M4.

**Extended Data Figure 5:**
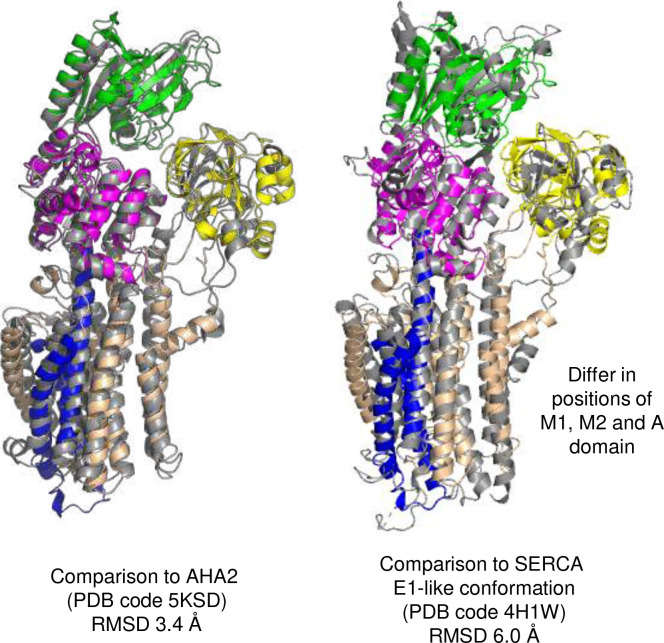
Alignment of the El-like·Mg conformation of MgtA (PDB code 9ME9) with previously reported structures of P-type ATPases. This conformation of MgtA is most similar to AHA2 and the E1-like conformation of SERCA.

In the presence of Mg^2+^, phosphate analogue, and the regulatory peptide MgtR, an E1-like conformation was observed in a mixed population with the E2-P conformation. In this E1-like conformation, the cytoplasmic domains were arranged in a more compact configuration compared to E2-P. Density associated with BeF_3_^−^ could not be detected in the maps for the E1-like conformation as expected, and an ambiguous density possibly associated with MgtR was found adjacent to transmembrane segment M3, but could not be modeled. Nonetheless, additional densities consistent with three bound Mg^2+^ ions were observed (Figure 3). The Mg^2+^ binding sites were similar to those reported previously ^16^, though the reported structures are distinct, particularly in the N-terminal region (M1-M4 and the A domain; Extended Data Figure 4). The E1-like conformation of MgtA was most like the yeast proton pump AHA2 ^18^ and SERCA in the E1-like conformation ^19^ based on structural comparison and overall RMSD (Extended Data Figure 5).

Two Mg^2+^ ions were coordinated by the cytoplasmic domains, one between the A and N domains and another between the A and P domains (sites 1 and 2, respectively; Figure 3). The first Mg^2+^ ion was found coordinated by Asp^434^ and Ile^521^ (backbone carbonyl) of the N domain and Asp^193^ and Glu^209^ of the A domain (Figure 3C). Similarly, the second Mg^2+^ ion was found coordinated by Thr^150^ (backbone carbonyl), Glu^162^, and Asp^180^ of the A domain and Asp^660^ of the P domain (Figure 3D). These Mg^2+^ ions were mainly coordinated by acidic residues that bridged the two domains, suggesting that they may be regulatory. They represent partial coordination spheres for Mg^2+^, which may only be occupied at high concentrations of Mg^2+^ (there are a total of 5 side chain oxygen atoms within 4 Å of the ion). This is consistent with the structure determination in the presence of 5 mM Mg^2+^, where MgtA activity is expected to be low (Figure 1). In site 1, Asp^193^ and Glu^209^ flank the TGES motif in the A domain of MgtA (residues Thr^202^-Gly^203^-Glu^204^-Ser^205^), and Asp^434^ is adjacent to Phe^438^, one of the residues involved in coordinating the adenine ring of ATP. This suggests a plausible regulatory mechanism for this site, where Mg^2+^ binding at high concentrations may modulate domain movements that are necessary for subsequent steps in the transport cycle.

**Extended Data Figure 6:**
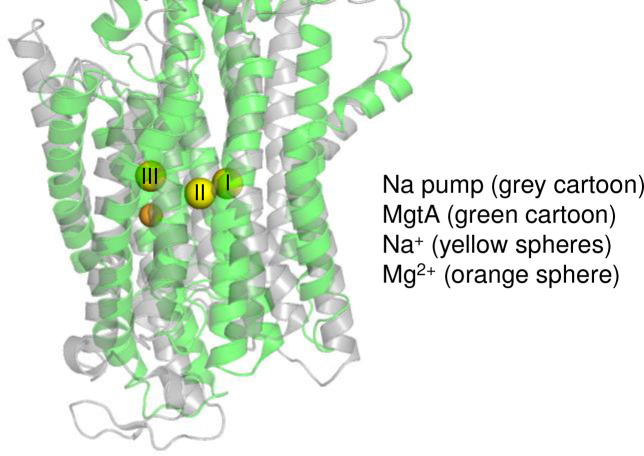
Alignment of the El-like·Mg conformation of MgtA (PDB code 9ME9) with previously reported structure of the Na^+^,K^+^-ATPase (PDB code 4HQJ). The Mg^2+^ ion bound in the transmembrane domain of MgtA is close to the site 3 sodium ion in Na^+^,K^+^-ATPase.

**Extended Data Figure 7:**
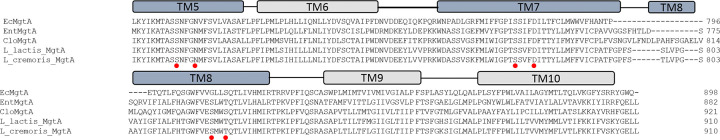
Limited sequence alignment of MgtA showing conservation of the Mg^2+^ binding site in the transmembrane segments M5 (Ser^702^ and Asn^706^), M7 (Ser^773^ and Asp^777^), and M8 (Ser^821^ and Thr^824^). The sequences for *E. coli*, *E. faecalis*, *C. difficile*, *L. lactis lactis*, and *L. lactis cremoris* are shown.

Transmembrane helices M5, M7, and M8 collectively enclose an additional density, which we (Figure 3E and Extended Data Figure 2D) and others ^16^ have assigned as a bound Mg^2+^ ion. The coordinating residues include Ser^702^ and Asn^706^ of M5, Ser^773^ and Asp^777^ of M7, and Ser^821^ and Thr^824^ of M8 (Figure 3E). Asn^706^, Asp^777^, and Ser^821^ are positioned “below” the Mg^2+^ ion on the periplasmic side of the binding site with the carbonyl and hydroxyl oxygen atoms in close proximity to the Mg^2+^ (2.0 to 3.6 Å). Ser^702^, Ser^773^, and Thr^824^ are positioned “above” the Mg^2+^ ion on the cytoplasmic side of the binding site with the hydroxyl oxygen atoms further from the ion (4.5 to 5.8 Å). This coordination around the Mg^2+^ ion, particularly on the cytoplasmic side of the membrane, suggests that the ion may be poised for release in the E1-like state. As stated above, the Mg^2+^ binding site is unique among the P-type ATPases and closest to the third sodium ion binding site in the Na^+^, K^+^-ATPase ^17^. However, the Mg^2+^ ion sits lower in the transmembrane domain and further from the cytoplasm compared to the third sodium ion (Extended Data Figure 6). In addition, sequence alignment of MgtA homologs across several bacterial species revealed a high degree of conservation in the Mg^2+^ coordinating residues, supporting their role in binding and transport activity (Extended Data Figure 7).

**Extended Data Figure 8:**
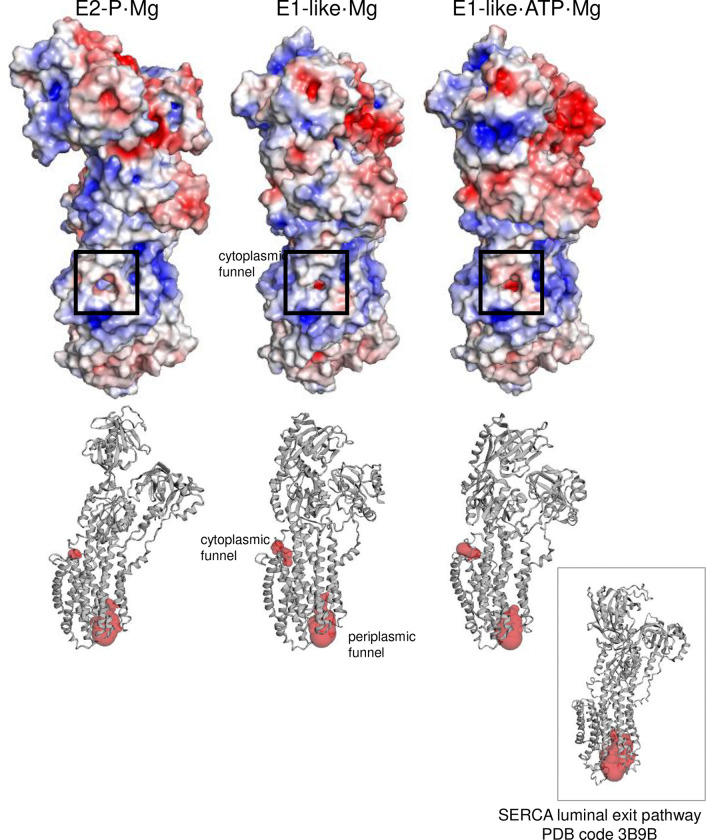
Electrostatic surfaces and CASTpFOLD predictions for the three main conformations of MgtA. Notice the absence of a cytoplasmic funnel in the E2-P·Mg conformaiton.

**Figure 4:**
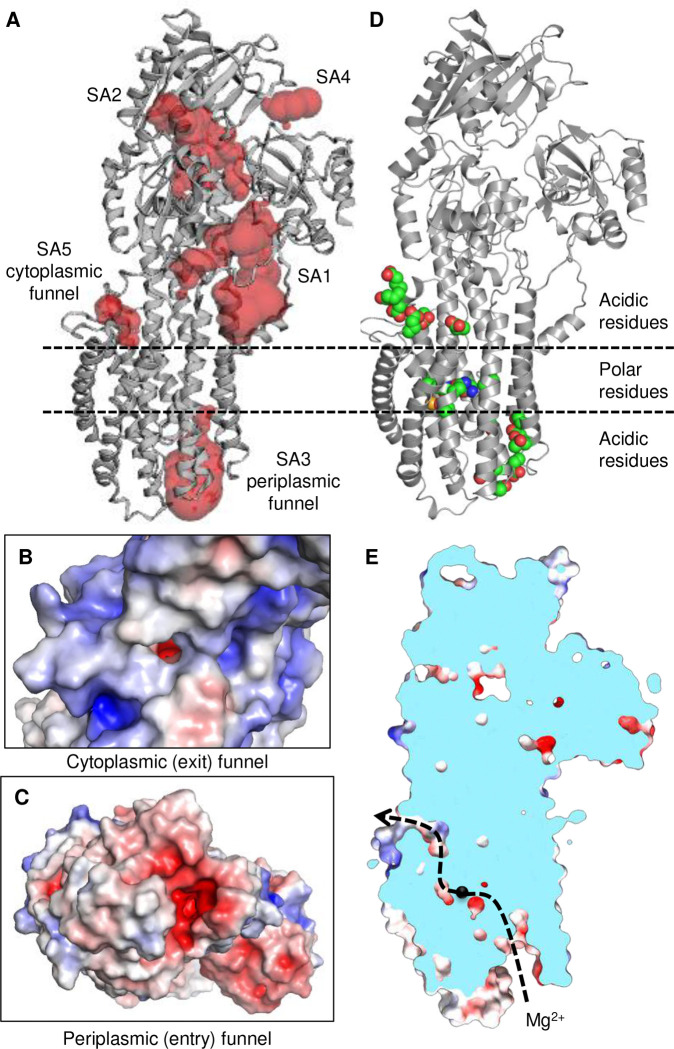
Potential entry and exit pathways for Mg^2+^ access to the transmembrane binding site. (**A**) CASTpFOLD prediction of surface-accessible cavities for MgtA in the E1-like·Mg state (top five sites SA1-SA5). (**B**) SA5 may form part of the cytoplasmic exit funnel. (**C**) SA3 may form part of the periplasmic entry funnel. (**D**) Acidic residues form the outer perimeters of the entry and exit funnels, and polar residues including the gating residue Glu^330^ are found in the transmembrane domain. These latter residues have been found to bind Mg^2+^ in the E1-like conformation of SERCA. (**E**) Solid surface rendering of MgtA and a cut-away view of the entry and exit funnels relative to the Mg^2+^ binding site (black sphere).

Like all P-type ATPases, MgtA follows the alternating access model of transport in which the Mg^2+^ binding site must be accessible to either the periplasmic side or the cytoplasmic side of the membrane, separated by intermediate occluded states. In an effort to identify access or release pathways to the transmembrane binding site for Mg^2+^, we used CASTpFOLD ^20^ to evaluate the surface topography of MgtA (Figure 4). The five largest surface-accessible volumes identified were the linker region (SA1; 1289 Å^3^), the nucleotide binding site (SA2; 836 Å^3^), a periplasmic funnel (SA3; 723 Å^3^), the gap between the A and N domains (SA4; 91 Å^3^), and a cytoplasmic funnel directly above the bound Mg^2+^ ion in the membrane (SA5; 62 Å^3^). The periplasmic funnel in MgtA is similar to the luminal calcium exit pathway in SERCA ^21,22^ (Extended Data Figure 8). Interestingly, the luminal pathway in SERCA contains a bound Mg^2+^ ion that interacts with the calcium-gating residue Glu^309^ and stabilizes the open exit pathway. This gating residue is conserved in MgtA (Glu^330^) and oriented toward the periplasmic funnel, suggesting that it could serve as a gating residue for Mg^2+^ transport. The cytoplasmic pathway in MgtA is similar to the C-terminal exit pathways proposed for proton counter-transport in SERCA and Na^+^, K^+^-ATPase ^23^. Thus, the periplasmic and cytoplasmic funnels of MgtA are potential entry and exit pathways for Mg^2+^, with acidic residues at the entrance of each funnel and a series of polar residues that bridge the gating residue Glu^330^ and the Mg^2+^ binding site (Figure 4D, E).

### E1-like·ATP·Mg conformation of MgtA

**Figure 5:**
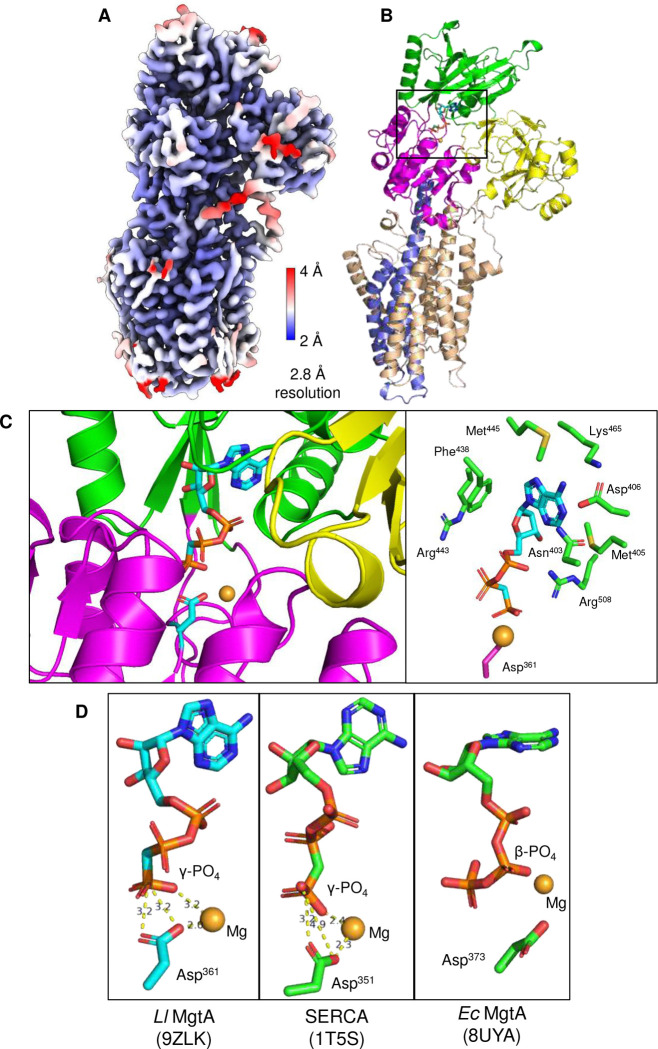
Cryo-EM reconstruction of MgtA in the E1-like·ATP·Mg state in the presence of Mg^2+^ and AMPPCP. (**A**) Cryo-EM map colored according to estimated resolution for a monomer. (**B**) Cartoon diagram of MgtA in the E1-like·ATO·Mg state colored according to the A (yellow), P (magenta), N (green), and transmembrane segments M5, M7, and M8 (blue). Based on PDB code 9ZLK (Table 1). (**C**) AMPPCP and a Mg^2+^ ion bound in the nucleotide binding pocket relative to the catalytic Asp^361^. The residues involved in coordinating nucleotide are shown. (D) The poise of nucleotide in MgtA compared to SERCA (PDB code 1T5S) with both appearing to be catalytically competent states. A previous structure of MgtA is also shown (PDB code 8UYA), which exhibits a very different mode of binding compared to our structure (PDB code 9ZLK). Density map threshold was set to 0.1 in ChimeraX (8.5σ).

To determine the structural effect of ATP on the conformational state of MgtA, structure determination was performed in the presence of Mg^2+^ and the non-hydrolyzable ATP analogue, AMPPCP. A reconstruction was obtained at 2.8 Å resolution revealing well-ordered cytoplasmic and transmembrane domains, and an additional density in the cytoplasmic domain that was attributable to bound AMPPCP and Mg^2+^ (Figure 5 and Extended Data Figure 2). An additional density was also observed in the transmembrane domain, consistent with the bound Mg^2+^ ion present in the E2-P and E1-like structures of MgtA. AMPPCP binding to MgtA was similar to what has been observed with SERCA ^24^. The ATP binding pocket contains residues that coordinate the adenine ring including Asn^403^ (2.6 Å), Phe^438^ (3.3 Å), and Lys^465^ (3.1 Å), as well as additional residues (Met^405^, Asp^406^, and Met^445^) that provide a complementary binding pocket (Figure 5C). A pair of arginine residues, Arg^443^ and Arg^508^, flank the phosphates and position the γ-phosphate and a Mg^2+^ ion in close proximity to the catalytic aspartate, Asp^361^ (Figure 5D). By comparison to SERCA, this appears to be a catalytically competent conformation of ATP binding to MgtA. In contrast, previous structures of *E. coli* MgtA revealed ATP bound with the β-phosphate proximal to the catalytic aspartate, Asp^373 16^.

### Lipid interactions with MgtA

**Figure 6:**
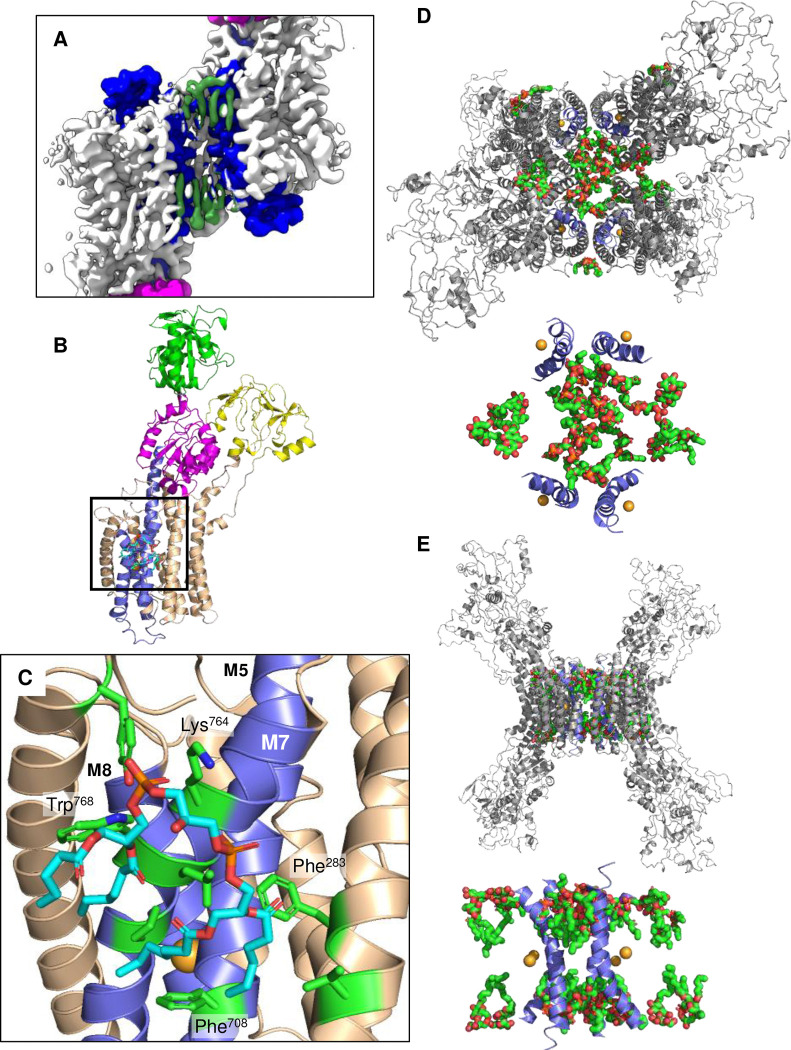
Lipids identified in cryo-EM maps of MgtA. (**A**) A specifically-bound cardiolpin molecule (green surface) in the E2-P·Mg conformation of MgtA (PDB code 9BYB). (**B**) Cartoon diagram of MgtA in the E2-P·Mg state colored according to the A (yellow), P (magenta), N (green), and transmembrane segments M5, M7, and M8 (blue). The cardiolipin binds to transmembrane segment M7 near the proposed exit pathway for Mg^2+^. (**C**) Residues of MgtA that contribute to cardiolipin binding, including Lys^764^ and Trp^768^ on M7. (**D**) and (**E**) Lipids and detergents modeled into additional densities identified in the antiparallel tetramer of MgtA (PDB code 9Q1E). Many of the lipids are proximal to M7 (blue helix) and the Mg^2+^ binding site (orange sphere) in the transmembrane domain.

In all reconstructions of MgtA, the transporter was surrounded by a density that was consistent with a DDM micelle (Supplementary Figure 9) and lipids that co-purified with MgtA (Figure 6). Lipid molecules could be modelled at select sites, many of which were consistent with a cardiolipin structure of four aliphatic tails and connected head group. Several well-ordered cardiolipins were observed adjacent to transmembrane helices of MgtA that coordinate the bound Mg^2+^ ion, particularly M7. In the reconstruction of MgtA in the E2-P conformation (PDB code 9BYB), a cardiolipin was identified adjacent to M7 on the cytoplasmic side of the membrane (Figure 6A, B). A lysine residue, Lys^764^ on M7, appears to interact with the negatively charged head group of the cardiolipin, while aromatic residues (Phe^283^, Phe^708^, and Trp^768^) support the interaction of the acyl chains (Figure 6C).

The lipid interactions were most apparent in the dimer and tetrameric reconstructions of MgtA, but were present in monomers as well. High-resolution cryo-EM reconstructions of the tetramer revealed well-defined densities, enabling the modeling of 17 lipids and 4 detergents into the density map (PDB code 9Q1E). Many of these densities were consistent with cardiolipin, though there may be ambiguity in modeling some of the densities. Nonetheless, a large number of lipids are present in the core of the tetramer and are completely surrounded by protein. While the tetramers are non-physiological arrangements of MgtA, transmembrane segment M7 faces the inner core of the tetramer. This places a high density of lipids and negatively charged head groups in close proximity to the Mg^2+^ binding site (Figure 6D, E) and the alleged exit pathway (Figure 4). The antiparallel arrangement of MgtA and the presence of lipids on both sides of the tetramer core suggest that the periplasmic and cytoplasmic surfaces are enriched in negatively charged lipids. Thus, the structural data appears to support prior observations that MgtA activity is enhanced by cardiolipin ^11^.

## DISCUSSION

### Mg^2+^ transport by MgtA

High-resolution structures of the bacterial P-type ATPase Mg^2+^ transporter, MgtA, were determined in three distinct conformational states: E2-P·Mg (Figure 2; 2.6 Å resolution), E1-like·Mg (Figure 3; 2.7 Å resolution), and E1-like·ATP·Mg (Figure 5; 2.8 Å resolution). These structures provide new insights into the conformational landscape of MgtA and the binding of particular substrates – Mg^2+^, phosphate, and ATP. E2-P·Mg is a new conformation of MgtA that appears to be a covalent aspartyl-phosphate complex of the enzyme with a Mg^2+^ counter-ion bound in the transmembrane domain. In this conformation, the Mg^2+^ ion is fully occluded by transmembrane helices M5, M7, and M8, as there is no clear exit pathway (Extended Data Figure 8). In the E1-like·Mg conformation of MgtA, phosphate is no longer bound and the Mg^2+^ ion remains coordinated by transmembrane helices M5, M7, and M8. A potential exit pathway to the cytoplasm appears, though the Mg^2+^ ion is still in an occluded state. Finally, the E1-like·ATP·Mg is a new catalytically-competent conformation of MgtA, where the exit pathway is still present and the Mg^2+^ ion remains occluded. In the alternating access model for membrane transport, the structures suggest that the E2-P·Mg conformation is an outward-facing occluded state with a potential entry channel facing the periplasm. The E1-like·Mg and the E1-like·ATP·Mg conformations appear to be inward-facing occluded states with the formation of a potential exit pathway facing the cytoplasm.

Overall, the structures reveal a possible pathway for Mg^2+^ transport by MgtA from the periplasm to the cytoplasm and the roles of particular residues. This pathway is remarkably similar to an ion pathway proposed for the Na^+^, K^+^-ATPase ^25^. In this scenario, Mg^2+^ enters through a negatively charged funnel formed by the N-terminal transmembrane helices M1, M2, M4, and M5 (Figure 4E). The Mg^2+^ ion encounters the gating residue, Glu^330^, and a series of conserved residues that are involved in Mg^2+^ binding by SERCA (PDB code 3W5B ^26^). Mg^2+^ may transiently interact with these residues, Leu^327^ and Glu^330^ (M4), Asn^714^ and Asn^717^ (M5), and Asn^742^ (M6), before moving to the transport site formed by transmembrane helices M5, M7, and M8 (Figure 3). A conformational change must then occur, likely corresponding to the formation of the E1~P intermediate of MgtA, which may open an exit channel to the cytoplasm formed by the C-terminal transmembrane helices M5, M7, and M8. Recall that the MgtA transport cycle occurs during starvation, where Mg^2+^ concentrations are extremely low. Negatively charged surfaces of the protein and lipid head groups may assist in transport by attracting Mg^2+^ ions to these regions. The proposed periplasmic entry pathway for Mg^2+^ is strongly negative, suggesting that it could attract Mg^2+^ ions from the periplasm even when concentrations are low. The proposed exit pathway is more neutral, though the loop between transmembrane helices M6 and M7 sits above the exit channel and contains several charged residues (Asp^747^, Glu^748^, and Glu^749^). Finally, MgtA appears to associate with lipids in the cryo-EM maps, many of which were recognizable as cardiolipin lipids. Cardiolipin is known to enhance MgtA activity ^11^, and the observed lipids were concentrated around transmembrane segment M7 and the bound Mg^2+^ ion (Figure 6). This suggests that the negatively charged head groups could be important for Mg^2+^ release.

## Conclusions

Structural intermediates along the Mg^2+^ transport pathway, as well as the mechanisms that regulate this process, have remained major gaps in our understanding of membrane transport and P-type ATPase function. Here, we present new structures of MgtA that uncover previously unrecognized features of ion transport by P-type ATPases. The transported Mg^2+^ ion occupies a distinct binding site within the transmembrane domain, with a plausible transport route that extends from a periplasmic entry funnel to a cytoplasmic exit funnel. These structures capture elusive states of the transport cycle, including a P-type ATPase bound to its elusive counter ion and a catalytically-competent, ATP-bound intermediate that remains counter ion-bound. In addition, they reveal new mechanisms by which transmembrane peptides and membrane lipids regulate transporter function, highlighting conserved modes of control that likely extend beyond the P-type ATPase family to the general mechanisms by which the membrane environment controls protein structure and function.

## MATERIALS and METHODS

### Expression of MgtA

*Escherichia coli* BL21 (DE3) pLysS cells were transformed with a pET vector encoding His-tagged MgtA variant from *Lactococcus lactis cremoris* and *Lactococcus lactis lactis* and plated on agar containing carbenicillin and chloramphenicol. Following overnight incubation, a single colony was inoculated into 50 mL of Luria-Bertani (LB) broth supplemented with 100 μg/mL carbenicillin and 30 μg/mL chloramphenicol and grown overnight at 220 rpm. The next day, 5 mL of the overnight culture was used to inoculate 1 L of LB medium containing 100 μg/mL carbenicillin. The culture was incubated at 37°C with shaking at 220 rpm until the optical density at 600 nm (OD_600_) reached 0.6. Protein expression was induced by the addition of 500 μL of 0.5 M isopropyl β-D-1-thiogalactopyranoside (IPTG), and the temperature was reduced to 18°C. The culture was incubated overnight with shaking at 200 rpm.

After 16 hours, cells were harvested by centrifugation at 5,000 × g for 15 minutes at 4°C. The resulting cell pellets were resuspended in buffer containing 2 mM EGTA, 5 mM NaF, 0.66 mM BeSO_4_, 20 mM HEPES (KOH), 50 mM LiCl, 5 mM MgCl_2_, and 20% glycerol. Cells were lysed using a sonicator set to 70% power for a total of 5 minutes, with 1-minute intervals. The crude lysate was clarified by centrifugation at 20,000 × g for 20 minutes at 4°C, and the resulting supernatant was subjected to ultracentrifugation at 100,000 × g for 1 hour. The membrane pellet was washed by homogenization in 50 mL of buffer to remove residual soluble proteins. All cell lysis and membrane isolation steps were performed at 4°C. Membrane fractions were aliquoted, flash-frozen in liquid nitrogen, and stored at −80°C until further use.

### Purification of MgtA

Membrane fractions (~10 mg/mL) were solubilized in 1% n-dodecyl-β-D-maltoside (DDM) and incubated at 4°C for 16 hours with continuous rotation. Insoluble material was removed by ultracentrifugation at 100,000 × g for 1 hour at 4°C. The solubilized membrane proteins were supplemented with imidazole to a final concentration of 10 mM and loaded onto a Ni-NTA affinity column three times at 4°C to enhance binding efficiency. The resin was washed with 10 column volumes of Buffer A (2 mM EGTA, 5 mM NaF, 0.66 mM BeSO_4_, 20 mM HEPES, 50 mM LiCl, 5 mM MgCl_2_, 0.1% DDM, 10 mM imidazole, and 20% glycerol). Bound MgtA was subsequently eluted using Buffer B (2 mM EGTA, 5 mM NaF, 0.66 mM BeSO_4_, 20 mM HEPES, 50 mM LiCl, 5 mM MgCl_2_, 0.1% DDM, 250 mM imidazole, and 20% glycerol). The eluted protein was concentrated to approximately 12 mg/mL using 100 kDa molecular weight cutoff Amicon Ultra Centrifugal Filters.

The concentrated protein solution was clarified by centrifugation at 30,000 × g for 30 minutes at 4°C before being subjected to size-exclusion chromatography (SEC) using a HiLoad 16/600 Superdex 200 PG column equilibrated with buffer (2 mM EGTA, 5 mM NaF, 0.66 mM BeSO_4_, 20 mM HEPES, 50 mM LiCl, 5 mM MgCl_2_, 0.01% DDM, and 5% glycerol). Gel filtration fractions containing purified MgtA were analyzed by sodium dodecyl sulfate-polyacrylamide gel electrophoresis (SDS-PAGE). The peak fractions were pooled and concentrated using 100 kDa molecular weight cutoff Amicon Ultra Centrifugal Filters to a final concentration of 10–12 mg/mL for further structural analyses.

### Expression and purification of MgtR

Recombinant MgtR was expressed and purified using techniques previously described ^27–30^ with the following alterations: MgtR sequence was inserted into a pMal vector with the sequence MNHSSDKIIA_10_LIFLLMSLLV_20_LLLALWQIVL_30_.

### Magnesium-dependent ATPase activity of MgtA

ATPase activity was measured for both detergent solubilised and reconstituted MgtA using the malachite green assay Pi ColorLockTM ALS according to manufacturer’s guidelines (Innova Biosciences). Purified MgtA was either used directly in the detergent-solubilized state as described above or reconstituted into proteoliposomes using established procedures ^31–33^. The magnesium-dependent ATPase activity assays were performed in 96-well microtiter plates at 22 °C in 50 mM HEPES-KOH buffer, pH 7.0, 50 mM KCl, 2 mM ATP. Free magnesium concentrations in the range of 1 to 100 μM were used. For detergent solubilised MgtA, 0.1 % DDM was added to the assay mixture. Reactions were started by the addition of MgtA, and after desired times of incubation, ALS mixture was added to terminate the reaction. Stabilizer was then added and incubated for 30 minutes, and the absorbance was measured at 625 nm. The V_max_ (maximal activity) and K_Ca_ (apparent calcium affinity) were determined based on non-linear least-squares fitting of the activity data to the Hill equation (Sigma Plot software, SPSS Inc., Chicago, IL). Errors were calculated as the standard error of the mean (SEM) for a minimum of four independent samples.

### Mass Photometry

Mass photometry was performed by preparing 6mg/mL MGTA and 6mg/mL MGTA + MGTR in buffer (2mM EGTA, 5mM NaF, 0.66mM BeSO_4_, 50mM LiCl, 5mM MgCl_2_, 20mM HEPES pH 7.5, 5% glycerol, 0.006% DDM) and diluting to 0.03 mg/mL with the same buffer. 1 μL of this diluted stock was added to 19 μL of buffer that was positioned on a glass coverslip mounted and focused on a Refeyn TwoMP Mass Photometer (Refeyn Ltd., Oxford, UK), yielding a final concentration of 1.5 μg/mL for MGTA and MGTA+MGTR. A movie of interference signals was recorded on a portion of the glass coverslip for 60 seconds. The movie was then converted into a ratiometric format using the Refeyn DiscoverMP software package to clearly identify points of interference. Interference events were then recorded and displayed in histogram format to display the distribution of interferometric mass-related events. These events were then fitted to a Gaussian peak using the DiscoverMP software and compared to a previously determined standard curve of BSA and Thyroglobulin. The mass profile of both MGTA and MGTA + MGTR in buffer are reported in kDa ± 1 S.D. (Supplementary Figure 1).

### Sample preparation of MGTA for Cryo-EM

For cryo-sample preparation, purified MGTA protein was concentrated to 6 mg/mL. Samples were then diluted to 2 mg/mL and centrifuged at 15 000 × RCF for 30 minutes at 4°C. Purified MGTA (3 μL) was then applied to glow-discharged Quantifoil R1.2/1.3 Cu 300 grids, blotted for 6.5 seconds, and vitrified in liquid ethane with a Thermo Fisher Scientific Vitrobot Mark IV (4°C, 100% humidity, blot force 0).

### Sample preparation of MGTA + MGTR for Cryo-EM

For cryo-sample preparation, peak fractions of purified MGTA protein were pooled and concentrated to 6 mg/mL. Samples were then diluted to 3 mg/mL with buffer containing 6 mg/mL MGTR and allowed to incubate at 4°C for 30 minutes. The mixture was centrifuged at 15,000 × RCF for 30 minutes at 4°C. 3 μL of MGTA+MGTR complex was then applied to glow-discharged Quantifoil R1.2/1.3 Ni 300, blotted for 7 seconds, and vitrified in liquid ethane with the Vitrobot Mark IV (4°C, 100% humidity, blot force 0).

### Cryo-EM of MGTA

Movies were collected on a Titan Krios G3i at 300 kV, outfitted with a K3 (Gatan) direct electron detector, using super-resolution mode. Movie collection was automated using EPU, where ~14,000 movies were collected. Super-resolution images were Fourier cropped to 0.788 Å/px for image processing. Each movie was collected with 45 frames with a total dose of 44 e^−^/Å^2^ at a defocus range of −0.8 μm to −2.0 μm. Movies and sample preparation was prepared on-site at the Pacific Northwest Cryo-EM Center (Portland, Oregon, USA).

### Cryo-EM of MGTA+MGTR

Movies were collected on a Titan Krios G3 at 300 kV outfitted with a Falcon 4i direct electron detector and Selectris-X energy filter. Movie collection was automated using EPU where ~20,000 electron event representations (EER) were collected. Pixel size used for data collection was 0.93 Å/px with an energy filter slit width of 20 eV. Each EER had 40 frames selected with a total dose of 45–50 e^−^/Å^2^ at a defocus range of −0.8 μm to −2.0 μm. Data collection and sample preparation were performed on-site at the Pacific Northwest Cryo-EM Center (Portland, Oregon, USA)

### Data processing of MGTA

The data processing pipeline can be visualized in supplemental figures outlined in Table 1. Data was processed using CryoSPARC v4.0 (Structura Biotechnology, Toronto, ON, Canada) processing suite. Gatan K3 or Falcon 4i super-resolution movies were Fourier cropped by one-half during Patch Motion Correction, resulting in motion-corrected exposures. CTF estimation of exposures was determined using Patch CTF Estimation. Exposures were manually curated and used for Blob Picking (80–320 Å blob diameter). 2–6 million particles were selected and subjected to several rounds of 2D Classification. Approximately 200,000 representative particles were then selected for ab initio reconstruction and homogeneous refinement, resulting in a better than 5 Å GSFSC map. This map was then subjected to several rounds of Non-Uniform Refinement and CTF Refinement until no further changes to GSFSC occurred. This data processing pipeline resulted in the reconstructions of MGTA listed in Table 1.

### Model Building of MGTA

A preliminary structural model was generated using Swiss-Model and ModelAngelo ^34^, employing a targeted homology approach with the crystal structure of a plasma membrane proton pump (PDB code 5KSD ^35^) and the nucleotide-binding domain of MgtA (PDB code 3GWI ^36^) as templates. The resulting model was segmented into distinct structural fragments using PyMOL; the A domain (residues 10–62 and 148–256), N domain (residues 373–541), P domain (residues 357–372 and 542–674), transmembrane segments M1 and M2 (residues 63–147), and transmembrane segments M3-M4 and M5-M10 (residues 257–356 and 675–910, respectively). These fragments were individually docked into the 3D cryo-EM map using UCSF ChimeraX ^37^. Following docking, flexible fitting was performed in Coot ^38^ to refine regions that did not align properly, particularly portions of the A-domain, which required manual adjustment. Poorly resolved regions were further refined using 3DFlex and the non-sharpened map to trace the backbone and connect structural segments. Subsequent real-space refinement was conducted using Phenix ^39^ (version 1.21.2_5419) to optimize the geometry and improve the model's accuracy.

The quality of the refined structure was assessed using MolProbity ^40^ for geometric validation. Structural visualization and figure generation were performed using UCSF Chimera and ChimeraX. The final fitted PDB models and cryo-EM maps have been deposited in the EMDB. Cryo-EM data collection and refinement statistics are summarized in Table 1, which lists the data processing supplementary figures and tables for each map and PDB model.

## Extended Data

**Extended Data Figure 9:**
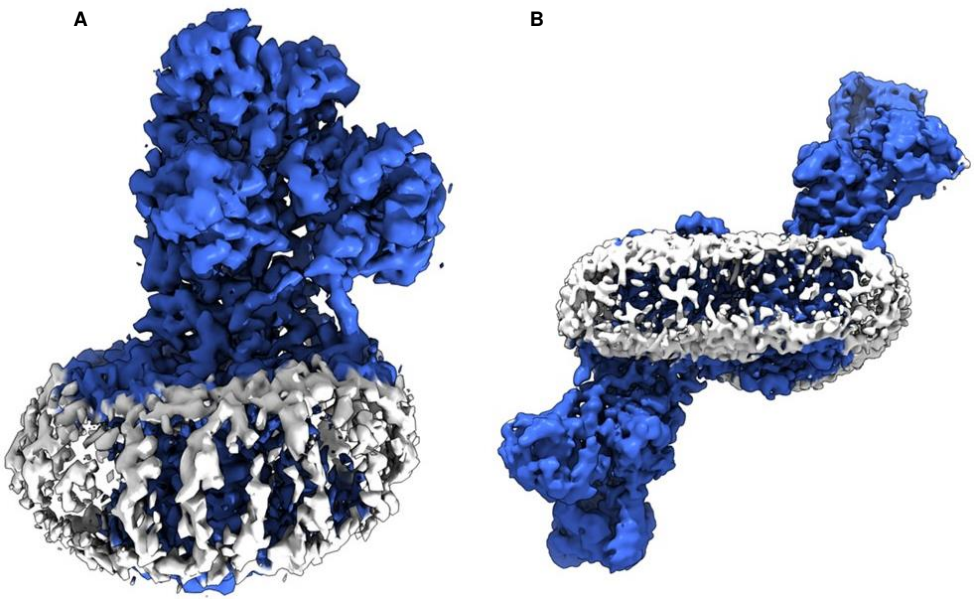
Representative detergent-lipid micelles associated with the reconstructions of MgtA monomers and dimers. The density maps are shown as a solid surface with a threshold of 0.25 in ChimeraX (10σ) for the monomer and 0.02 in ChimeraX (5σ) for the dimer. The MgtA density is in blue and the micelle density in grey.

**Extended Data Table 1:** Cryo-EM data collection, processing, refinement, and model building and validation statistics.

	9MQM MGTA E2-P EMDB-48534 (Monomer)	9MT7 MgtA E2-P EMDB-48602 (Monomer)	9Q6O MgtA E2-P EMDB-72280 (Monomer)	9BYB MgtA E2-P EMDB-45025 (Dimer)	9N5J MgtA E2-P EMDB-48923 (Dimer)	9Q1E MgtA E2-P EMDB-72125 (Tetramer)	9ME9 MgtA E1-like EMDB-48191 (Monomer)	9N3V MgtA E2-P, E1-like EMDB-48855 (Dimer)	9NHZ MgtA 3 E2-P, 1 E1-like EMDB-49450 (Tetramer)	9ZLK MgtA E1-Like EMDB-74405 (Monomer)
**Data collection and processing**										
Microscope	Titan Krios G3i	Titan Krios G3i	Titan Krios G3i	Titan Krios G3i	Titan Krios G3i	Titan Krios G3i	Titan Krios G3i	Titan Krios G3i	Titan Krios G3i	Titan Krios G3i
Detector	Falcon 4i	Falcon 4i	Falcon 4i	K3	Falcon 4i	Falcon 4i	K3	Falcon 4i	Falcon 4i	Falcon 4i
Energy Filter	Selectris X, 20eV	Selectris X, 20eV	Selectris X, 20eV	none	Selectris X, 20eV	Selectris X, 20eV	Selectris X, 20eV	Selectris X, 20eV	Selectris X, 20eV	Selectris X, 20eV
Magnification	130,000x	130,000x	130,000x	29,000x	130,000x	130,000x	130,000x	130,000x	130,000x	130,000x
Voltage (keV)	300	300	300	300	300	300	300	300	300	300
Electron exposure (e^−^/Å^2^)	45	45	50	44	45	45	45	45	45	50
Defocus range (μm)	−0.8 to −2.0	−0.8 to −2.0	−0.8 to −2.0	−0.8 to −2.0	−0.8 to −2.0	−0.8 to −2.0	−0.8 to −2.0	−0.8 to −2.0	−0.8 to −2.0	−0.8 to −2.0
Pixel size (Å)	0.93	0.93	0.93	0.788	0.93	0.93	0.93	0.93	0.93	0.93
Symmetry imposed	C1	C1	C1	C1	C1	C1	C1	C1	C1	C1
Initial particle images (no.)	7,760,668	7,760,668	4,824,248	2,308,732	3,931,186	6,552,046	6,949,991	3,931,186	6,552,046	11,028,750
Final particle images (no.)	650,314	411,840	180,870	174305	252,574	86,188	128,354	194,186	105,182	577,182
Map resolution (Å)	3.1	3.23	2.65	3.29	2.59	2.73	3.2	2.65	2.69	2.79
FSC threshold	0.143	0.143	0.143	0.143	0.143	0.143	0.143	0.143	0.143	0.143
Map resolution range (Å)_	2.0 – 9.0	2.0 – 10.2	2.0 – 6.5	3.10 – 8.44	2.0 – 7.8	2.0 – 8.1	3.5 – 8.9	2.0 – 6.7	2.0 – 8.4	1.3 – 8.9
**Refinement**										
Initial model used (PDB code)	5KSD, 3GWI	5KSD	5KSD	5KSD	5KSD	5KSD	5KSD	5KSD	5KSD	5KSD
Model resolution (Å)	3.4	3.4	2.8	3.6	2.70	3.1	3.70	3.0	3.1	2.79
FSC threshold	0.5	0.5	0.5	0.5	0.5	0.5	0.5	0.5	0.5	0.5
Map sharpening *B* factor (Å^2^)	−106.7	−103.3	−66.2	−88.6	−69.0	−49.8	−86.6	−62.6	−50.4	−89.4
**Model composition**										
Non-hvdrogen atoms	6831	6531	6908	13666	13686	28466	6833	13683	27354	7006
Protein residues	888	888	891	1764	1778	3556	888	1778	3556	897
Ligands	Mg^+2^	Mg^+2^	Mg^+2^, BeF_3_	Mg^+2^, CDL	Mg^+2^, BeF_3_	Mg^+2^, BeF_3_, 4DDM, 17CDL	Mg^+2^	Mg^+2^, BeF_3_	Mg^+2^	Mg^+2^, K^+^, AMPPCP
**RMSDs**										
Protein	141.0	128.49	70.41	141.91	150.00	202.02	148.3	140.15	186.94	101.67
Ligand	74.7	56.24	62.97	102.9	103.21	160.90	125.9	122.04	50.0	148.90
**B factors (Å^2^)**										
Bond lengths (Å) (no. >4σ)	0.004	0.003	0.004	0.004	0.007	0.008	0.003	0.005	0.005	0.007
Bond angles (°) (no. >4σ)	0.741	0.708	0.979	0.849	0.652	1.194	0.509	1.106	1.083	1.053
**Validation**										
MolProbity score	2.44	2.36	1.72	2.70	2.23	2.41	1.51	2.17	2.43	1.94
Clashscore	14.13	11.96	6.68	15.60	11.37	12.46	7.06	10.83	14.88	5.75
Poor rotamers (%)	1.13	2.36	1.83	4.09	0.45	2.45	1.44	2.62	2.29	4.24
**Ramachandran plot**										
Favored (%)	90.52	91.99	97.18	90.51	94.02	91.46	98.42	95.72	92.14	97.21
Allowed (%)	5.35	7.67	2.71	8.75	5.52	7.78	1.35	4.11	7.10	2.79
Disallowed (%)	1.13	0.34	0.11	0.74	0.45	0.76	0.23	0.17	0.76	0.00
**Correlation model versus data**										
CC (mask, volume)	0.84 (.84)	0.70 (069)	0.88 (0.87)	0.84 (0.82)	0.84 (0.83)	0.75 (0.75)	0.74 (0.75)	0.84 (0.84)	0.82 (0.82)	0.82 (0.82)
CC (ligands)	0.88	0.79	0.61	0.69	0.79	0.36	0.63	0.68	0.79	0.47

## Figures and Tables

**Figure 7:**
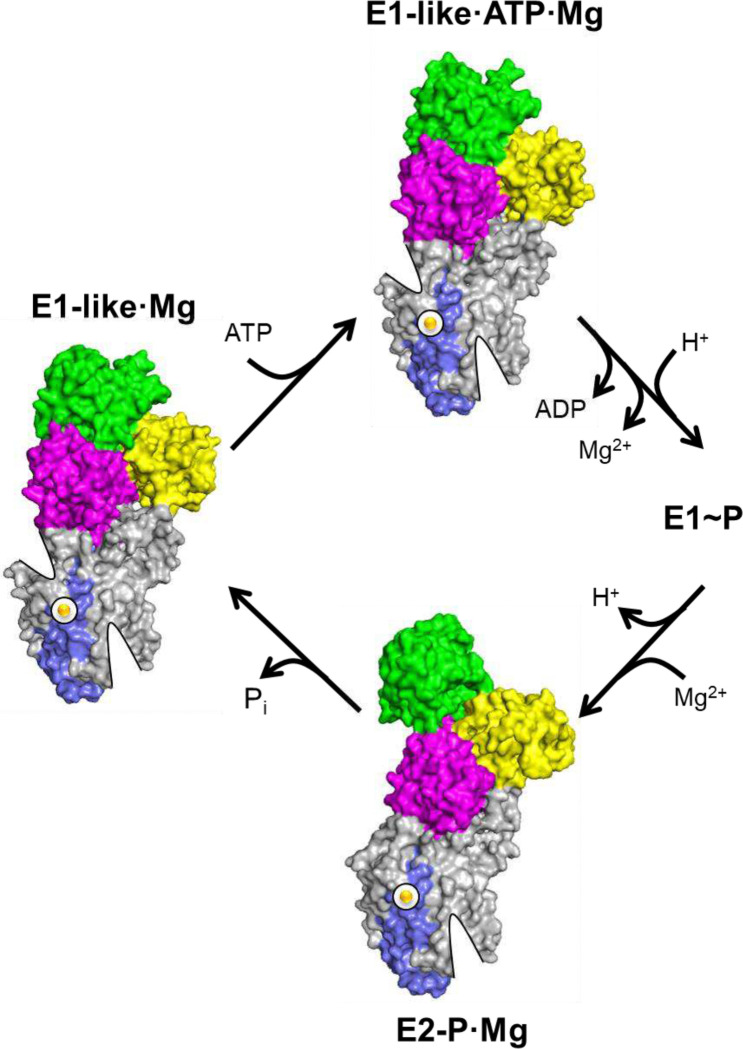
Simplified model for the transport cycle of MgtA based on the E2-P·Mg, E1-like·Mg, and E1-like·ATP·Mg conformations. Mg^2+^ binds from the periplasm to form the E2-P·Mg occluded state. The TGES loop then hydrolyzes the phosphate and inorganic phosphate is released (P_i_). This promotes the E1-like·Mg conformation of MgtA with a partially open exit pathway for Mg^2+^ release to the cytoplasm. This conformation is in a favorable state for ATP binding, where ATP promotes an E1-like·ATP·Mg with Mg^2+^ still bound. ATP binds in a catalytically competent conformation and the partially open exit pathway remains. We propose that aspartyl-phosphate formation leads to the formation of a high-energy E1~P state, which triggers opening of the exit pathway and release of Mg^2+^ to the cytoplasm in exchange for a proton (H^+^). The E1~P conformation is an occluded state for the bound proton (H^+^), which is poised to release the energy captured from ATP, bind Mg^2+^ again, and transition to the occluded E2-P·Mg conformation.

**Table 1:** Summary of MgtA structures determined in the present study.

PDB code	Conformational state	Ligands bound	Oligomeric state	Resolution	Extended Data Information
**Cryo-EM of MgtA in the presence of Mg^2+^ and BeF_3_^−^**					
9MQM	E2-P	Mg^2+^	monomer	3.1 Å	Ext Fig 2A Ext Table 1
9MT7	E2-P	Mg^2+^	monomer	3.2 Å	Ext Fig 2B Ext Table 1
9Q6O	E2-P	Mg^2+^, BeF_3_^−^	monomer	2.7 Å	Ext Fig 2C, D Ext Table 1
9BYB	E2-P	Mg^2+^, cardiolipin	dimer	3.3 Å	Ext Fig 2E Ext Table 1
9N5J	E2-P	Mg^2+^, BeF_3_^−^	dimer	2.6 Å	Ext Fig 2F, G Ext Table 1
9Q1E	E2-P	Mg^2+^, BeF_3_^−^ 4 DDM, 17 CDL	tetramer	2.7 Å	Ext Fig 2H Ext Table 1
**Cryo-EM of MgtA in the presence of Mg^2+^, BeF_3_^−^, and MgtR**					
9ME9	E1-like	Mg^2+^	monomer	3.2 Å	Ext Fig 2I Ext Table 1
9N3V	E2-P, E1-like	Mg^2+^, BeF_3_^−^	mixed dimer	2.7 Å	Ext Fig 2J Ext Table 1
9NHZ	Three E2-P, one E1-like	Mg^2+^	mixed tetramer	2.7 Å	Ext Fig 2K Ext Table 1
**Cryo-EM of MgtA in the presence of Mg^2+^ and AMPPCP**					
9ZLK	E1-like	Mg^2+^, K^+^, AMPPCP	monomer	2.8 Å	Ext Fig 2L Ext Table 1

## Data Availability

Cryo-EM maps and atomic coordinates were deposited into the EMDB and RCSB PDB, respectively. The final unsharpened cryo-EM maps have been deposited in the Electron Microscopy Data Bank (EMDB) under the following accession codes: EMD-48534 (PDB: 9MQM), EMD-48602 (PDB: 9MT7), EMD-72280 (PDB: 9Q6O), EMD-45025 (PDB: 9BYB), EMD-48923 (PDB: 9N5J), EMD-72125 (PDB: 9Q1E), EMD-48191 (PDB: 9ME9), EMDB-48855 (PDB: 9N3V), EMDB-49450 (PDB: 9NHZ), and EMDB-74405 (PDB: 9ZLK). Additionally, an X-ray crystal structure has been deposited in the Protein Data Bank (PDB) under the accession code 9EJN.
